# Development of primary care quality indicators for chronic obstructive pulmonary disease using a Delphi-derived method

**DOI:** 10.1038/s41533-022-00276-w

**Published:** 2022-03-18

**Authors:** Sigrid Dewaele, Steve Van den Bulck, Lien Gerne, Bert Vaes

**Affiliations:** Department of Public Health and Primary Care, Academic Center For General Practice, KU Leuven, Kapucijnenvoer 7 blok H, Box 7001, 3000 Leuven, Belgium

**Keywords:** Chronic obstructive pulmonary disease, Health policy

## Abstract

High-quality care for patients with COPD is necessary. To achieve quality improvement in primary care, the general practitioner and the electronic health record (EHR) play an important role. The aim of this study was to develop a set of evidence-based and EHR extractable quality indicators (QIs) to measure and improve the quality of COPD primary care. We composed a multidisciplinary expert panel of 12 members, including patients, and used a RAND-modified Delphi method. The SMART principle was applied to select recommendations and QIs from international guidelines as well as existing sets of QIs, and these recommendations and QIs were added to an individual written questionnaire. Based on the median score, prioritization and degree of agreement, the recommendations and QIs were rated as having a high, uncertain or low potential to measure the quality of COPD primary care and were then discussed in an online consensus meeting for inclusion or exclusion. After a final validation, a core set of recommendations was translated into QIs. From 37 recommendations, obtained out of 10 international guidelines, and 5 existing indicators, a core set of 18 recommendations and 2 QIs was derived after the rating procedure. The expert panel added one new recommendation. Together, the recommendations and QIs were translated and merged into a final set of 21 QIs. Our study developed a set of 21 evidence-based and EHR-extractable QIs for COPD in primary care. These indicators can be used in an automated quality assessment to measure and improve the quality of COPD primary care.

## Introduction

COPD is a major disease burden in terms of morbidity, disability, mortality, and health-care cost^1–4^. Although COPD is a progressive and life-threatening disease, it is manageable. With appropriate management, most people with COPD can achieve good symptom control and quality of life, as well as reduced risk of other related conditions.

High-quality care for patients with COPD is important, mainly due to serious consequences and the growing number of affected people^5^. Multiple international COPD guidelines for primary care exist and are routinely updated^6^. However, general practitioners do not always adjust their practice according to the latest recommendations to ensure the most appropriate care for patients with COPD^7–10^. To measure these limitations and to improve the quality of COPD primary care, quality indicators (QIs) are needed^11^. Quality assessment focuses on health-care outcomes, structures and processes, and coded data in the electronic health record (EHR) can be used to evaluate the quality of care^12–16^. Moreover, EHR-extractable QIs drastically increase the number of patients whose quality of care can be assessed^12^. As a result, feedback to health-care professionals can make them reflect on their own practice and assist them in improving the quality of care^17^.

Although QIs for COPD care are available in the literature, few cover all aspects of COPD care^18–20^. In addition, they are not specifically intended for primary care or can be extracted out of the EHR. The aim of this study was therefore to develop an up-to-date set of evidence-based and EHR extractable QIs that cover all aspects of COPD primary care, thus enabling the monitoring and improvement of the quality of COPD primary care.

## Methods

We conducted our study from December 2019 to April 2021.

### Study design

To develop QIs for COPD in primary care, we used a RAND-modified Delphi method^11,13,21,22^. The following steps were included: (1) Selection of recommendations and existing QIs from evidence-based guidelines; (2) rating procedure by an expert panel in three rounds: (a) individual written questionnaire, (b) online consensus meeting, and (c) final appraisal; and (3) translation of the recommendations into QIs.

### Ethical approval

The study was approved on December 9, 2019, by the Research Ethics Committee UZ/KU Leuven with reference MP012007.

### Panel selection

Multiple health-care providers and patients with COPD were contacted via e-mail or phone call: pulmonologists of three hospitals via e-mail invitations, general practitioners via local general practitioner circles and patients with COPD via a non-profit patient association. The selection of possible participants was based on their expertize with COPD. All patients needed to have the diagnosis of COPD for >10 years and all professionals needed to treat patients with COPD on a daily basis to ensure this inclusion criterion was met. In total, 12 participants were included to construct a multidisciplinary panel based on profession, patient involvement, and expertize. They received information about the aim of the study and the methodology in an informed consent letter. We asked them to sign the consent form before participation.

### Selection of recommendations and QIs from evidence-based guidelines

**Fig. 1:**
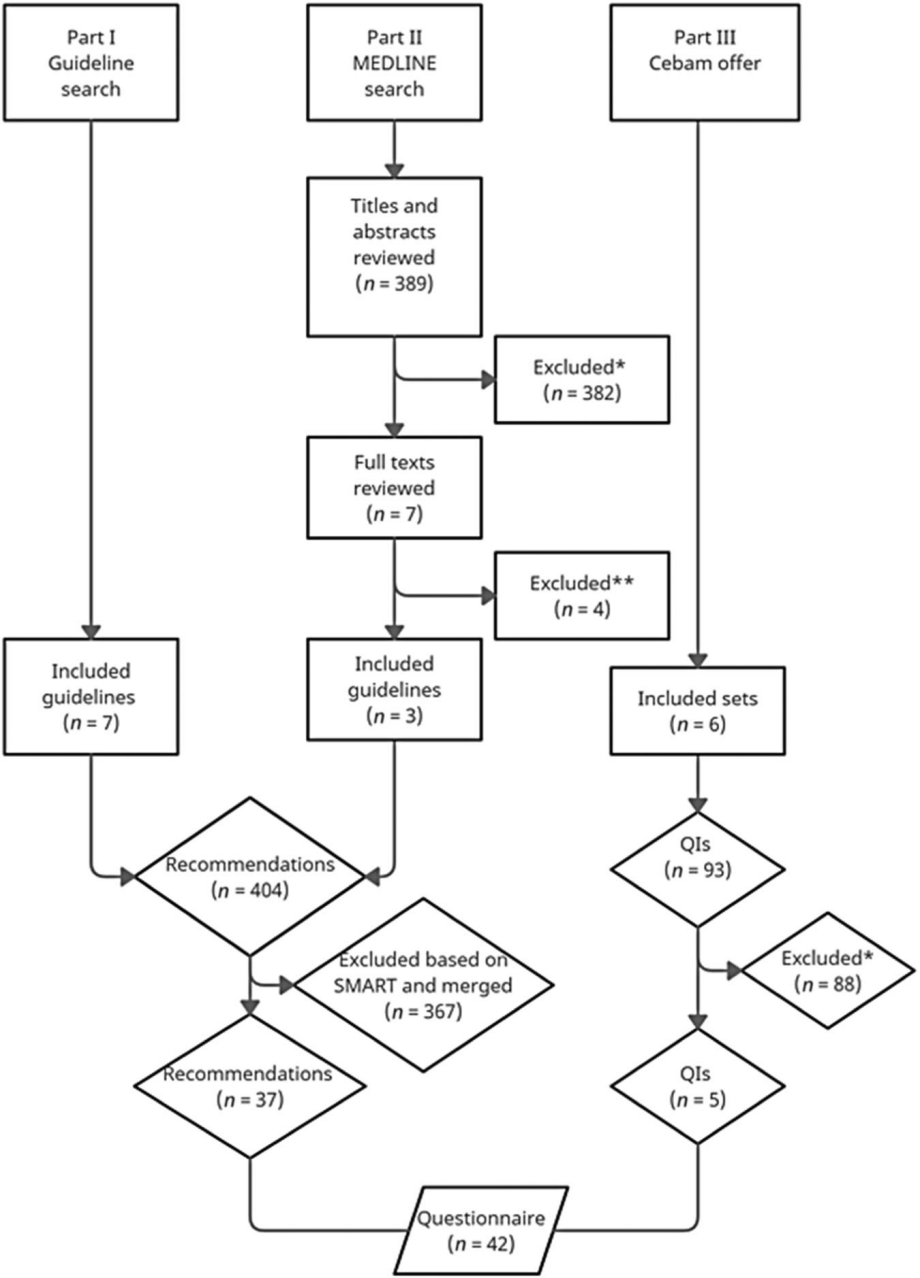
Flowchart of selection of recommendations and QIs from evidence-based guidelines. *Titles, abstracts, and quality indicators excluded for one of the following reasons: published before 2012, no English or Dutch, no full text access, incorrect subject (e.g., lung cancer screening), incorrect target users (e.g., pulmonologists), no guidelines or duplicates. **Full texts excluded because three did not sufficiently meet quality criteria and one was a duplicate.

First, we searched for national and international guidelines on the World Wide Web and by reference tracking^20,23–28^. These guidelines had to be evidence-based and developed by internationally renowned institutions. Second, a literature review using the electronic database MEDLINE was performed until January 4, 2020, for additional guidelines or recommendation statements (see Fig. 1). The search strategy combined MeSH terms and free text: “chronic obstructive pulmonary disease” OR “chronic bronchitis” OR “pulmonary emphysema” OR “COPD” AND “guideline” OR “standard of care” OR “clinical protocols” OR “health planning guidelines”. One investigator (S.D.) screened all titles and abstracts to exclude irrelevant references. References were retained if they were clinical guidelines or recommendation statements aimed at clinicians in primary care in which all aspects of the care for COPD, including definition, screening, diagnosis, treatment, management, comorbidities, referral, follow-up, pulmonary rehabilitation or palliative and end-of-life care, was recommended. Then, full texts of all potentially relevant guidelines or recommendation statements were read and reviewed by two investigators (S.D. and L.G.) for their eligibility. In case of disagreement, a third author (S. VdB.) was consulted.

Third, a list of existing sets of QIs for COPD developed by international health organizations was offered by the Belgian Centre for Evidence-Based Medicine (www.cebam.be).

The entire selection process was based on language (English and Dutch) and year of publication (after 2012). Furthermore, an instrument developed by the Appraisal of Guidelines for Research and Evaluation (AGREE) Collaboration was used independently by two investigators (S.D. and L.G.) to ensure that only guidelines of high quality were implemented^29^. Guidelines with scores of <50% were excluded.

One investigator (S.D.) exhaustively extracted all recommendations from the included guidelines and listed them in (sub)categories regarding their domain (see Supplementary Table 2). Thereafter, QIs for COPD from the Cebam list were added if they met the aforementioned eligibility criteria and if they were not already covered. Then, the EHR extractability and the applicability to primary care of the recommendations and QIs were judged based on the SMART principle^30^. Finally, the selected recommendations and QIs were included in a questionnaire that was provided to the panel (see Supplementary Table 3). An alternative copy of the questionnaire was provided to the patients, excluding the recommendations specifically aimed at health-care providers.

### Rating procedure

The questionnaire was sent by e-mail to the panel members accompanied by a clear explanation of the objectives of the study and specific instructions to fill in the questionnaire (round a). They were asked to score each recommendation and QI by giving a number on a 9-point Likert scale, with 1 being the lowest score and 9 the highest score for capability to measure the quality of COPD primary care. If a panel member was unable to assess the recommendation or QI, then it was possible to designate “not assessable”. The EHR extractability could also be denoted. Per category, the panel members were asked to prioritize the recommendations and QIs with a maximum top-5 rank. In addition, panel members were given the opportunity to motivate their score, to give remarks or to suggest new recommendations.

All scores and remarks were entered into an Excel database and analyzed. The recommendations and QIs were assigned as having a high, uncertain or low potential to measure the quality of COPD primary care. These three criteria were based on the median Likert scale score, prioritization and degree of agreement among the panel members. First, the median Likert scale score was the calculation of the median score for each recommendation and QI. Hereby, non-assessable scores were omitted from the calculation. Second, prioritization meant that, for example, in a top 3, the first one in the ranking received 3 points, the second received 2 points and the last received 1 point. These points were then converted into percentages using the number of panel members that scored that recommendation or QI and the highest possible score. Third, agreement was defined as ≥70% of the panel members giving a median Likert score of ≥7, and disagreement was defined as ≥30% of the panel members giving a median Likert score ≥7 AND ≥ 30% giving ≤3. Finally, we refer to Table 1 for the categorization into high, uncertain or low potential to measure the quality of COPD primary care.

**Table 1:** Classifying recommendations and QIs into categories of high, uncertain and low potential.

Category of potential	Criteria of analysis
HIGH	MEDIAN ≥ 7 AND PRIORITIZATION ≥ 20% AND AGREEMENT
UNCERTAIN	MEDIAN ≥ 7 AND PRIORITIZATION 1–20% AND AGREEMENT OR
	MEDIAN < 7 AND PRIORITIZATION ≥ 20% AND AGREEMENT OR
	MEDIAN < 7 AND PRIORITIZATION ≥ 20% AND DISAGREEMENT
LOW	MEDIAN < 7 AND PRIORITIZATION < 20% AND AGREEMENT OR
	MEDIAN < 7 AND PRIORITIZATION < 20% AND DISAGREEMENT OR
	MEDIAN ≥ 7 AND PRIORITIZATION ≥ 20% AND DISAGREEMENT OR
	MEDIAN ≥ 7 AND PRIORITIZATION 1–20% AND DISAGREEMENT

The online consensus meeting was held via video conferencing (round b). Prior to the meeting, the panel members were informed of the results from the questionnaire in a feedback report. The report contained for each recommendation and QI their individual score, the median score, percentage of prioritization and agreement. Additionally, the assigned criterion of high, uncertain or low potential for quality of COPD care measurement was indicated. At the meeting, panel members first reviewed the recommendations and QIs with a low or high potential for exclusion or inclusion, respectively. They were encouraged to openly discuss their views of each recommendation and QI to reach consensus. Panel members were also allowed to modify the included recommendations and QIs and create new recommendations, keeping in mind the SMART principle to evaluate EHR extractability and appropriateness in primary care. Finally, the recommendations and QIs marked as uncertain potential were discussed until agreement was reached to exclude or include, possibly with modification. If reaching consensus was elusive, then a formal vote was held, and recommendations and QIs with >50% panel member support were included.

The list of included recommendations and QIs obtained from the consensus meeting was sent by e-mail to the panel members for their final appraisal (round c).

### Translation of recommendations into QIs

In the last step, the recommendations were translated into QIs as a percentage. For example, the recommendation “COPD patients should be offered annual vaccination with the influenza vaccine” was translated into “The percentage of patients with COPD who are vaccinated with an influenza vaccine annually”.

## Results

### Panel selection

A multidisciplinary expert panel (*n* = 12) consisted of four pulmonologists, three general practitioners, one physiotherapist, one nurse and three COPD patients. The panel members mainly originated from two regions in Flanders (province of West Flanders and province of Flemish Brabant). The health-care providers worked either in hospitals or in general practices. We did not account for factors such as age, gender, education level, or disease severity.

### Selection of recommendations and QIs from evidence-based guidelines

The selection process for recommendations and QIs from evidence-based guidelines is visualized in Fig. 1. First, seven guidelines were selected. Second, 389 references were identified through the database search. In total, 382 irrelevant references were excluded based on title and abstract. Subsequently, seven full texts were retrieved and assessed for eligibility. Of these, four references were excluded: three did not meet the quality criteria, and one was a duplicate. Together, ten guidelines fulfilled the eligibility criteria^20,23–28,31–33^. Third, six sets of existing QIs (*n* = 93) were added.

Initially, 404 recommendations were extracted from the included guidelines. They were classified into ten main categories (definition, screening, diagnosis, treatment, management, comorbidities, referral, follow-up, pulmonary rehabilitation and end-of-life care). After applying the SMART principle and merging similar recommendations, 37 recommendations were used in the questionnaire. In addition, five QIs from the sets were added to the questionnaire.

### Rating procedure

The rating procedure included three rounds. All panelists (12/12) completed the individual written questionnaire (round a). The questionnaire for health-care providers consisted of 37 recommendations and five QIs. The COPD patients were asked to fill in the same set without the pharmacological part (18 recommendations). Based on the median score, prioritization and degree of agreement, 27 recommendations were assigned as high, ten as low and five as uncertain potential to measure the quality of COPD primary care (see Supplementary Table 1).

Thereafter, eight panel members (three pulmonologists, three general practitioners, one nurse, and one physiotherapist) participated in the online consensus meeting (round b). One out of ten recommendations and QIs with low potential was included. Then, 23 out of 27 high-potential recommendations and QIs were retained. No recommendation or QI of uncertain potential was accepted. In total, 20 recommendations and four QIs were included, and 17 recommendations and one QI were excluded (see Supplementary Table 1).

Although the SMART principle was used, the expert panel decided to not exclude two recommendations and one preexisting QI that are currently not extractable from the EHR. Because of their importance for the quality of COPD primary care, some panel members proposed having these recommendations and QI made codable by computer experts in the nearest future.

The expert panel emphasized a maximum duration of seven days for both oral corticosteroids and antibiotics to treat an acute exacerbation. Therefore, at the consensus meeting, the panel’s pulmonologists introduced one new recommendation expressing the maximum duration of antibiotic treatment in case of an acute exacerbation. Finally, this resulted in a list of 21 recommendations and four QIs.

After the consensus meeting, the core set of 21 recommendations and four QIs was sent by e-mail to the expert panel for final appraisal (round c). The four panel members who were unable to attend the consensus meeting agreed without any adjustments. All panel experts approved the final set.

### Translation of recommendations into QIs

The recommendations were finally translated into 19 QIs, and two preexisting QIs were added, resulting in a final set of 21 QIs. The final set (Table 2) of QIs was made up of two QIs on definition, two QIs on screening, two QIs on diagnosis, three QIs on prevention and nonpharmacological treatment, nine QIs on pharmacological treatment, of which one was added by the expert panel, two QIs on referral and one QI on end-of-life care.

**Table 2:** Final set of 21 quality indicators (QIs) for COPD.

Definition	
1a	Percentage of patients diagnosed with COPD and GOLD 1, or FEV_1_ ≥ 80%
1b	Percentage of patients diagnosed with COPD and GOLD 2, or 50% ≤ FEV_1_ < 80%
1c	Percentage of patients diagnosed with COPD and GOLD 3, or 30% ≤ FEV_1_ < 50%
1d	Percentage of patients diagnosed with COPD and GOLD 4, or FEV_1_ < 30%
2a	Percentage of patients diagnosed with COPD in group A
2b	Percentage of patients diagnosed with COPD in group B
2c	Percentage of patients diagnosed with COPD in group C
2d	Percentage of patients diagnosed with COPD in group D
Screening	
3	Percentage of patients older than 40 years presenting with dyspnea, chronic cough or sputum production, a history of recurrent lower respiratory tract infections and/or a history of exposure to COPD risk factors (e.g., tobacco smoking), who received spirometry
4	Percentage of adults, including pregnant women, whose smoking status is assessed
Diagnosis and physical examination	
5	Percentage of patients with a diagnosis of COPD who had their diagnosis confirmed by spirometry after bronchodilation (FEV_1_/FVC < 0.7)
6	Percentage of patients with COPD GOLD 4 and a measurement of the oxygen saturation in the last 12 months
Prevention and nonpharmacological treatment	
7	Percentage of patients with COPD who are vaccinated with an influenza vaccine annually
8	Percentage of patients with COPD who are vaccinated with a pneumococcal vaccine
9	Percentage of patients with COPD in whom degree of physical activity is determined
Pharmacological treatment	
10	Percentage of patients with COPD who started with inhaled bronchodilators
11	Percentage of patients with COPD who were prescribed a LABA or LAMA, except COPD patients with only occasional dyspnea and for immediate relief of symptoms in patients already on long-acting bronchodilators for maintenance therapy
12	Percentage of patients with COPD in group A who are treated with a bronchodilator; this can be either a short- or a long-acting bronchodilator
13	Percentage of patients with COPD in group B who are treated with a long-acting bronchodilator
14	Percentage of patients with COPD in group C who are treated with a LAMA
15a	Percentage of patients with further exacerbations on LABA/LAMA who are escalated to LABA/LAMA/ICS if blood eosinophils ≥ 100 cells/μL
15b	Percentage of patients with further exacerbations on LABA/LAMA and nonsmoker, who are added azithromycin if blood eosinophils < 100 cells/μL
16	Percentage of patients with an acute COPD exacerbation who are treated with a SABA with or without a SAMA
17	Percentage of patients with an acute COPD exacerbation who are treated with prednisone 40 mg per day for maximum 7 days
Referral	
18	Percentage of patients with COPD who are younger than 40 years and were referred to a pulmonologist
19	Percentage of patients with COPD and a referral to a pulmonologist if peripheral oxygen saturation is < 92% when stable, or haemoptysis, or > 2 thoracic infections a year, or mMRC > 2
End-of-life care	
20	Percentage of patients with COPD and FEV_1_ ≤ 30% and starting with long-term oxygen therapy for whom advance care planning is determined
Added by the expert panel	
21	Percentage of patients with an acute COPD exacerbation who are treated with an antibiotic for maximum 7 days, except COPD patients with bronchiectasis for whom the duration of antibiotic use is maximum 10 days

*COPD* chronic obstructive pulmonary disease, *GOLD* global initiative for chronic obstructive lung disease, *FEV*_*1*_ forced expiratory volume in one second, *FEV*_*1*_*/FVC* forced expiratory volume in one second/forced vital capacity, *LABA* long-acting beta_2_-agonist, *LAMA* long-acting muscarinic antagonist, *ICS* inhaled corticosteroids, *SABA* short-acting beta_2_-agonist, *SAMA* short-acting muscarinic antagonist, *mMRC* modified Medical Research Council dyspnea questionnaire.

The two recommendations and one preexisting QI that are currently not extractable from the EHR could not yet be translated into measurable QIs and were listed separately from the final set (Table 3).

**Table 3:** Recommendations and QI on COPD care, belonging to the core set of recommendations and QIs as defined by the expert panel, but not yet translatable into measurable QIs.

Prevention	
1	Smoking cessation is recommended for all COPD patients
Follow-up	
2	COPD patients should be monitored every 6 months. The CAT score should be used to detect symptoms related to COPD
3	Percentage of patients with COPD with an indication of the number of exacerbations in the last 12 months and in whom the degree of functioning using the mMRC score is determined

*COPD* chronic obstructive pulmonary disease, *CAT* COPD Assessment Test, *mMRC* modified Medical Research Council dyspnea questionnaire.

## Discussion

This study, using a RAND-modified Delphi method, developed a set of 21 evidence-based and EHR extractable QIs to assess the quality of COPD primary care. The set contains different aspects: definition, screening, diagnosis, prevention, nonpharmacological treatment, pharmacological treatment, referral and end-of-life care. In addition, these QIs can be used in an automated audit and feedback intervention as a framework to facilitate and improve the quality assessment of COPD primary care. Furthermore, this set of QIs is based on ten international guidelines and approved by a multidisciplinary expert panel including patients.

In the current study, a number of indications for referral were drawn up by the pulmonologists. Moreover, a prominent role in the follow-up and end-of-life care of COPD patients was assigned to the general practitioner. To date, these two categories have received too little interest. Other studies have already mentioned that a large proportion of patients with COPD receive inappropriate end-of-life care. Timely initiation of care planning could lead to a reduction in avoidable inappropriate end-of-life care^34,35^. No recommendations on pulmonary rehabilitation were selected. However, this does not mean that pulmonary rehabilitation is not important, but our expert panel stated that this therapy is mostly initiated by a pulmonologist. In Belgium, pulmonary rehabilitation services are usually connected to hospitals involving a team of different medical and paramedical disciplines. Therefore, these recommendations were not specific for primary care in Belgium. In addition, to date, the initiation of pulmonary rehabilitation is not extractable out of the EHR in Belgium and thus these recommendations were also not SMART applicable.

This study differs from the available literature in two main respects. First, in previous literature, for example the ACOVE-3 study or the HEDIS program, QIs were specifically aimed to specialists or only applied to elders^19,36^. Additionally, COPD QIs developed by large health organizations, such as the National Institute for Health and Care Excellence (NICE), did not cover the broad spectrum of COPD primary care^20,23^. Also, the focus of the existing QIs was mainly on diagnosis and management. Therefore, we designed a set of QIs exclusively for general practitioners, including all aspects of the medical process. Second, in our study, much attention was given to the EHR extractability of the QIs, which was not stated in another study^18^. One of our panel members was an expert in the use of the EHR as a member of the Intego network (www.intego.be). However, two recommendations and one preexisting QI were retained because of their great importance but are currently not extractable from Belgian EHR systems. More specifically, the registration of smoking cessation advice and functioning scores (CAT and mMRC) were estimated to be very important. This shortcoming in EHR extractability should be resolved in the near future so that these recommendations and QI should not be regarded as inappropriate.

The current study can serve as a first step to assess and improve the quality of COPD primary care. Automated extraction from the EHR will enhance the usability of these QIs. As this depends on coded data, challenges facing EHR systems are to transform uncoded data into structured data for subsequent analyses. Therefore, new methodologies for full integration of data and unification in EHR systems are required to optimize data management and extraction^16^. The continuous development of EHRs holds promise that quality assessment will grow and lead to improvements in COPD health-care. Additionally, to assess the implementation of these QIs, they must be validated by operational testing. A clinical practice test in multiple EHR systems is the best way to judge the validity of our QIs before integrating them in an automated quality measurement. This set is currently being tested in daily practice in Belgium with regard to validity and reliability.

One of the strengths of this study is the use of a multidisciplinary expert panel representing different disciplines involved in COPD care from diverse settings and regions within Flanders. In addition, our study paid attention to the patient’s point of view by including patients in the expert panel, according to other studies describing the development of QIs^11,13,21^. This panel diversity allowed us to take into account all aspects of COPD care. Furthermore, we used a robust methodology (RAND-modified Delphi method) to develop the QIs for COPD^37^ and applied the SMART principle, which has proven its usability for the development of QIs^13,21,38^. Because of the broad international base, this study has high validity and generalizability. Therefore, the final set of QIs can be used to evaluate quality assessment across countries, but the EHR extractability needs to be confirmed when these QIs would be used in other countries^11^. Also, QIs may be associated with the health-care system in which they were developed. In Belgium, patients have free choice of health-care professionals and have direct access to them. Hence, for example, QIs for referral may differ between countries.

However, there are some limitations. Our panel did not include COPD educators or pharmacists, and it is known that another constitution of the panel could have led to a different set of QIs^39^. None of the patients were able to participate in the meeting, which took place online instead of face-to-face. Therefore, the decisions to include or exclude in this round may be biased in favor of those who attended the meeting. Nonetheless, this bias was minimized by giving the entire panel the opportunity to give their final appraisal by e-mail. A limitation of using the RAND-modified Delphi method is the loss of participant anonymity in the voting process during the consensus meeting^39,40^. However, a group consensus meeting allows panel experts to exchange important information, for example, clarification of recommendations or reasons for (dis)agreement. Additionally, seeking clarification helps to reach consensus or to generate an alternative recommendation.

In summary, this study developed a set of 21 evidence-based and EHR-extractable QIs for COPD in general practice using a RAND-modified Delphi method. These indicators cover different aspects of COPD primary care, including definition, screening, diagnosis, prevention, nonpharmacological treatment, pharmacological treatment, referral and end-of-life care. In addition, this set enables an automated audit and feedback intervention to assess and improve the quality of COPD primary care.

### Reporting summary

Further information on research design is available in the Nature Research Reporting Summary linked to this article.

## Supplementary information

Supplementary Data

Reporting Summary

## Data Availability

Raw data were generated at the KU Leuven facility. De-identified data that support the results of this study are available within the paper and its supplementary files.

## References

[1] Lozano R (2012). Global and regional mortality from 235 causes of death for 20 age groups in 1990 and 2010: a systematic analysis for the Global Burden of Disease Study 2010. Lancet.

[2] Vos T (2012). Years lived with disability (YLDs) for 1160 sequelae of 289 diseases and injuries 1990-2010: a systematic analysis for the Global Burden of Disease Study 2010. Lancet.

[3] Lõpez-Campos JL, Tan W, Soriano JB (2016). Global burden of COPD. Respirology.

[4] Gershon AS, Warner L, Cascagnette P, Victor JC, To T (2011). Lifetime risk of developing chronic obstructive pulmonary disease: A longitudinal population study. Lancet.

[5] Singh, D. et al. Global strategy for the diagnosis, management, and prevention of chronic obstructive lung disease: the GOLD science committee report 2019. *Eur. Respir. J*. **53**, 1900164 (2019). 10.1183/13993003.00164-2019 30846476

[6] Miravitlles M (2016). A review of national guidelines for management of COPD in Europe. Eur. Respir. J..

[7] Rodrigues C (2019). Does practice follow evidence-based guidelines? Adherence to GOLD guidelines in Portugal. Pulmonology.

[8] Hsieh MJ (2018). The impact of 2011 and 2017 Global Initiative for Chronic Obstructive Pulmonary Disease (GOLD) guidelines on allocation and pharmacological management of patients with COPD in Taiwan: Taiwan Obstructive Lung Disease (TOLD) study. Int. J. COPD.

[9] Bosse G (2011). Adherence to Guideline-based Standard Operating Procedures in Pre-hospital Emergency Patients with Chronic Obstructive Pulmonary Disease. J. Int. Med. Res..

[10] Bourbeau J (2008). Les modes de pratique pour la prise en charge de la maladie pulmonaire obstructive chronique en première ligne: L’étude CAGE. Can. Respir. J..

[11] Kötter T, Blozik E, Scherer M (2012). Methods for the guideline-based development of quality indicators-a systematic review. Implement. Sci.

[12] Campanella P (2016). The impact of electronic health records on healthcare quality: A systematic review and meta-analysis. Eur. J. Public Health.

[13] Van den Bulck SA (2020). Development of quality indicators for type 2 diabetes, extractable from the electronic health record of the general physician. A rand-modified Delphi method. Prim. Care Diabetes.

[14] Campbell SM, Braspenning J, Hutchinson A, Marshall MN (2003). Improving the quality of health care: Research methods used in developing and applying quality indicators in primary care. Br. Med. J..

[15] Mainz J (2003). Defining and classifying clinical indicators for quality improvement. Int. J. Qual. Heal. Care.

[16] Coppersmith NA, Sarkar IN, Chen ES (2019). Quality informatics: the convergence of healthcare data, analytics, and clinical excellence. Appl. Clin. Inform..

[17] Ivers, N. et al. Audit and feedback: effects on professional practice and healthcare outcomes. *Cochrane Database Syst. Rev*. **2012**, CD000259 (2012). 10.1002/14651858.CD000259.pub3 PMC11338587 22696318

[18] Gershon AS (2019). Development of quality indicators for chronic obstructive pulmonary disease (COPD): a modified RAND appropriateness method. *Can*. J. Respir. Crit. Care Sleep. Med..

[19] Kleerup E (2007). Quality indicators for the care of chronic obstructive pulmonary disease in vulnerable elders. J. Am. Geriatr. Soc..

[20] National Institute for Health and Care Excellence*.* Chronic obstructive pulmonary disease in adults: NICE quality standard. National Institute for Health and Care Excellence*,* 2016).

[21] Van Den Bulck SA (2020). Developing quality indicators for Chronic Kidney Disease in primary care, extractable from the Electronic Medical Record. A Rand-modified Delphi method. BMC Nephrol..

[22] Fitch, K. et al. The RAND/UCLA Appropriateness Method User’s Manual. 10.1016/j.jacc.2005.08.030 (2001).

[23] National Institute for Health and Care Excellence*.* Chronic obstructive pulmonary disease in over 16s: diagnosis and management. (National Institute for Health and Care Excellence, 2018). 31211541

[24] Global Initiative for Chronic Obstructive Lung Disease (GOLD). *Global Strategy for the Diagnosis, Management, and Prevention of Chronic Obstructive Pulmonary Disease 2019 Report*. (Global Initiative for Chronic Obstructive Lung Disease (GOLD), 2019) 10.1016/j.arbr.2019.06.014.

[25] NHG-Standaard COPD (M26). (Nederlands Huisartsen Genootschap (NHG), 2015).

[26] Zorgstandaard COPD. (Long Alliantie Nederland (LAN), 2016).

[27] Chronic obstructive pulmonary disease (COPD). (Duodecim Medical Publications Ltd, 2019).

[28] Lung Foundation Australia and the Thoracic Society of Australia and New Zealand*. The COPD-X Plan: Australian and New Zealand Guidelines for the management of Chronic Obstructive Pulmonary Disease 2017*. 10.5694/mja17.00686 (Lung Foundation Australia and the Thoracic Society of Australia and New Zealand, 2017).

[29] Brouwers MC (2010). AGREE II: Advancing guideline development, reporting and evaluation in health care. CMAJ.

[30] Tichelaar J (2016). A ‘SMART’ way to determine treatment goals in pharmacotherapy education. Br. J. Clin. Pharmacol..

[31] Stolz D (2018). Diagnosis, prevention and treatment of stable COPD and acute exacerbations of COPD: The Swiss Recommendations 2018. Respiration.

[32] Lim TK (2018). Ministry of health clinical practice guidelines: chronic obstructive pulmonary disease. Singap. Med. J..

[33] Siu AL (2016). Screening for chronic obstructive pulmonary disease US preventive services task force recommendation statement. JAMA - J. Am. Med. Assoc..

[34] De Schreye R (2018). Appropriateness of end-of-life care in people dying from COPD. Applying quality indicators on linked administrative databases. J. Pain. Symptom Manag..

[35] Vermylen JH, Szmuilowicz E, Kalhan R (2015). Palliative care in COPD: an unmet area for quality improvement. Int. J. COPD.

[36] NCQA*. Required HEDIS and CAHPS Measures for HEDIS Reporting Year 2020*. ncqa.org. (2021).

[37] Hsu C-C, Sandford BA (2007). The delphi technique: making sense of consensus. Pract. Assess. Res. Eval..

[38] Smets M (2018). Defining quality indicators for heart failure in general practice. Acta Cardiol..

[39] Jandhyala R (2020). Delphi, non-RAND modified Delphi, RAND/UCLA appropriateness method and a novel group awareness and consensus methodology for consensus measurement: a systematic literature review. Curr. Med. Res. Opin..

[40] Eubank BH (2016). Using the modified Delphi method to establish clinical consensus for the diagnosis and treatment of patients with rotator cuff pathology. BMC Med. Res. Methodol..

