# Supplementary material for: Development of primary care quality indicators for chronic obstructive pulmonary disease using a Delphi-derived method
Source: NPJ Prim Care Respir Med. 2022 Mar 18;32:12. doi: 10.1038/s41533-022-00276-w (PMC8933430; doi:10.1038/s41533-022-00276-w)
Supplement: Supplementary file 1 — Supplementary Data [file 41533_2022_276_MOESM1_ESM.pdf]

Supplementary Table 1

Results of the rating procedure

	Recommendation or QI	Round a				Round b
		Median score	Prioritization (%)	Degree of agreement	Category of potential	Included or excluded
1	For all COPD patients, a classification of severity of airflow limitation is indicated as follows (in patients with FEV1/FVC < 0.7): GOLD 1, mild, if FEV1 ≥ 80% predicted; GOLD 2, moderate, if 50% ≤ FEV1 < 80% predicted; GOLD 3, severe, if 30% ≤ FEV1 < 50% predicted and GOLD 4, very severe, if FEV1 < 30% predicted	8	67	Agreement	HIGH	Included
2	For all COPD patients, the assessment of symptoms and history of moderate and severe exacerbations results in a letter from A to D and should be recorded. This allows clinicians to initiate a treatment plan.	8	83	Agreement	HIGH	Included
3	COPD should be considered in any patient who has dyspnoea (progressive, on exertion or persistent), chronic cough or sputum production, a history of recurrent lower respiratory tract infections and/or a history of exposure to risk factors (e.g., tobacco smoking).	8,5	92	Agreement	HIGH	Included
4	Screening spirometry in the general asymptomatic population is not recommended.	6,5	39	No agreement	LOW	Excluded
5	Clinicians should ask all adults, including pregnant women, about tobacco use.	9	69	Agreement	HIGH	Included
6	% COPD patients with registration BMI and 'unwanted weight loss' in the last twelve months	7	55	No agreement	LOW	Excluded
7	% COPD patients GOLD 4 and a measurement of the oxygen saturation in the last twelve months	8	72	Agreement	HIGH	Included
8	Spirometry is required to make the diagnosis. The presence of a post-bronchodilator FEV1/FVC < 0.70 confirms persistent airflow limitation.	8	73	Agreement	HIGH	Included
9	Spirometry is required to make the diagnosis. A FEV1/FVC < 5th percentile of the reference population (or z < -1.65) after bronchodilation is consistent with COPD.	8	53	Agreement	HIGH	Excluded
10	In case of abnormal spirometry, repeat the test after 6 weeks, so that the patient is recovered from a possibly first presented exacerbation of COPD.	6	50	No agreement	LOW	Excluded
11	Smoking cessation is recommended for all COPD patients.	9	95	Agreement	HIGH	Included
12	All patients with COPD should have an annual influenza vaccination.	9	72	Agreement	HIGH	Included
13	All patients ≥ 65 years with COPD and all younger patients with significant comorbid conditions (including chronic heart or lung disease) should receive the pneumococcal vaccination: the PCV13 and PPSV23.	8,5	53	Agreement	HIGH	Included
14	Supplemental oxygen therapy should be prescribed to stable COPD patients if Pa02 ≤ 55 mmHg or SaO2 < 88%.	8	38	Agreement	HIGH	Excluded
15	% COPD patients in whom degree of physical activity is determined	8	42	Agreement	HIGH	Included
16	For patients with COPD, inhaled bronchodilators are preferred over oral bronchodilators. Bronchodilators are recommended as the initial treatment for all COPD groups.	8	73	Agreement	HIGH	Included

17	For patients with COPD, LABAs and LAMAs are preferred over short-acting agents except for patients with only occasional dyspnoea, and for immediate relief of symptoms in patients already on long-acting bronchodilators for maintenance therapy.	8	60	Agreement	HIGH	Included
18	Rescue short-acting bronchodilators should be prescribed to all patients for immediate symptom relief.	7	18	Agreement	UNCERTAIN	Excluded
19	All group A patients should be offered a short- or a long-acting bronchodilator.	6,5	9	No agreement	LOW	Included
20	For group B, initial therapy should consist of a LABA or a LAMA. Patients with persistent breathlessness should be escalated to a LABA/LAMA combination.	8	31	Agreement	HIGH	Included
21	For group C patients, starting therapy with a LAMA is recommended.	8	20	Agreement	HIGH	Included
22	For patients in group C, a second long-acting bronchodilator is the preferred treatment option if exacerbations persist.	7,5	3	Agreement	UNCERTAIN	Excluded
23	Patients in group D should be started on a LABA/LAMA combination, guided by the level of symptoms (e.g. CAT > 20).	8	17	Agreement	UNCERTAIN	Excluded
24	For patients with persistent exacerbations on long-acting bronchodilator monotherapy, escalation to either LABA/LAMA or LABA/ICS is recommended.	8	14	Agreement	UNCERTAIN	Excluded
25	In patients who develop further exacerbations on LABA/LAMA therapy two alternative pathways are suggested: escalation to LABA/LAMA/ICS if blood eosinophil counts ≥ 100 cells/µL or add roflumilast or azithromycin if blood eosinophil < 100 cells/µL.	7,5	20	Agreement	HIGH	Included
26	In patients who develop further exacerbations on LABA/ICS therapy, escalation to triple therapy by adding a LAMA is recommended.	8	6	Agreement	UNCERTAIN	Excluded
27	Before starting azithromycin, ensure the patient has had: an electrocardiogram (ECG) to rule out prolonged QT interval and baseline liver function tests. Review prophylactic azithromycin after the first 3 months, and then at least every 6 months.	7	86	Agreement	HIGH	Excluded
28	Theophylline is not recommended unless other long-term treatment bronchodilators are unavailable or unaffordable.	6,5	52	No agreement	LOW	Excluded
29	Mucolytic drug therapy should be considered in patients with a chronic productive cough and should be continued if there is symptomatic improvement.	6,5	62	No agreement	LOW	Excluded
30	To treat an acute exacerbation, a SABA with or without a SAMA should be used as initial bronchodilator. For example, increased doses of salbutamol 4-8 puffs via a metered dose inhaler and spacer every 3-4 hours should be used.	8	93	Agreement	HIGH	Included
31	To treat an acute exacerbation of COPD, methylxanthines should not be used.	7,5	39	No agreement	LOW	Excluded
32	To manage an exacerbation of COPD with a significant increase in breathlessness which interferes with daily activities, offer 30 mg oral prednisone daily. Glucocorticosteroid treatment should not be given for more than 5-7 days.	8	75	Agreement	HIGH	Included

33	For exacerbations with signs and symptoms of infection (increased volume and change in colour of sputum or fever), antibiotics should be offered. First-line agents include oral amoxicillin or doxycycline for 5-7 days.	7	43	No agreement	LOW	Excluded
34	Patients under the age of 40 with COPD should be referred to a pulmonologist.	8,5	79	Agreement	HIGH	Included
35	Referral is indicated in the following circumstances:<br>- SpO2 < 92% when stable<br>- Haemoptysis<br>- Frequent chest infections (i.e., more than annually)	8	73	Agreement	HIGH	Included
36	COPD patients should be followed up every 3-6 months. The CAT score should be used at each visit to track symptoms related to COPD.	7	71	Agreement	HIGH	Included
37	Routine yearly chest X-rays are not required.	6	32	No agreement	LOW	Excluded
38	% COPD patients with an indication of the number of exacerbations in the last twelve months and an assessment of the dyspnoea using the mMRC score	8	79	Agreement	HIGH	Included
39	% COPD patients in whom the degree of functioning using the mMRC score is determined	8	69	Agreement	HIGH	Included
40	Pulmonary rehabilitation is indicated in all COPD patients with relevant symptoms and/or a high risk for exacerbations.	8	83	Agreement	HIGH	Excluded
41	Patients hospitalized for a COPD exacerbation, initiate a pulmonary rehabilitation program within 4 weeks of discharge.	7	67	No agreement	LOW	Excluded
42	COPD patients with FEV1 ≤ 30% and starting on long-term oxygen therapy are candidates for end-of-life discussion and Advance Care Planning.	9	100	Agreement	HIGH	Included

Supplementary Table 2

	Exhaustive list of recommendations				
	Recommendation	Source	Year	Included (IN), excluded (EX) or merged (ME)	Evidence grading (if included or merged)
**1**	**Definition**				
1.1	Classification of airflow limitation severity in COPD (based on post-bronchodilator FEV1 and in patients with FEV1/FVC < 0.7): GOLD 1 if FEV1 ≥ 80% predicted; GOLD 2 if 50% ≤ FEV1 < 80% predicted; GOLD 3 if 30% ≤ FEV1 < 50% predicted and GOLD 4 if FEV1 < 30% predicted	GOLD<br>SwRC	2019<br>2018	IN	/<br>/
1.2	The assessment of symptoms/risk of exacerbations results in a letter (groups A to D). This allows clinicians to initiate a treatment plan.	GOLD<br>MoHS	2019<br>2018	IN	/<br>Grade D, Level 4
1.3	Indicate the severity of COPD is based on the degree of airway obstruction and also on the health problems experienced by the patient (symptoms, limitations, exacerbations and nutritional status):<br>Slight if none of the following criteria and moderate if ≥ 1 of the following criteria: MRC ≥ 3 or CCQ ≥ 2; ≥ 2 exacerbations per year treated with oral corticosteroids or ≥ 1 hospitalization due to COPD; FEV1 after bronchodilation < 50% of predicted or < 1.5L absolute or progressive lung function loss (for example, ↓ FEV1 > 150 mL/year) over 3 years or more (≥ 3 measurements); unwanted weight loss > 5%/month or > 10%/6 months, or reduced nutritional status (BMI < 21), without other explanation;<br>Severe if intensive supervision is generally required in the second or third line (for example by means of multidisciplinary rehabilitation) in order to achieve the treatment objectives	NHG	2015	EX	

1.4	Patients are classified according to burden of disease: Slight disease burden: Every patient with COPD who, according to the assessment, no longer meets the criteria for further analysis. This concerns patients with FEV1> 50% of predicted, without severe symptoms or limitations due to dyspnoea (MRC <3), without serious adaptation problems, without reduced nutritional status, without frequent exacerbations and for whom the burden of disease is only slightly influenced by comorbidity. Moderate disease burden: Any patient with COPD who, according to the assessment, meets the criteria for further analysis, whereby treatment close to home is possible (with or without an exercise program, for example), but where infrequent, more extensive monitoring (and possibly adjustment of the treatment) in the second line is necessary. This group benefits most from 'shared' care. Severe disease burden: Any patient with COPD who, according to assessment, meets the criteria for further analysis, requiring intensive support in the second or third line (for example, by means of multidisciplinary rehabilitation).	LAN	2016	EX	
1.5	Severity grading of COPD based on spirometry alone is of marginal clinical value. Severe and often rapidly progressive disease is suggested by abundant subjective symptoms, recurrent exacerbations (at least 2/year) and FEV1 less than 50% of the reference value.	FiMSD	2019	EX	
1.6	Assessing the severity of COPD should take into account lung function, history of exacerbations and comorbid conditions	COPD-X	2017	EX	
**2**	**Screening**				
2.1	COPD should be considered in any patient who has dyspnea, chronic cough or sputum production, a history of recurrent lower respiratory tract infections and/or a history of exposure to risk factors for the disease.	GOLD	2019	ME	/
2.2	Screening spirometry in the general asymptomatic population is not recommended	MoHS<br>USPSTF	2018<br>2016	IN	Grade D, Level 4<br>Grade D
2.3	For patients over 40 years of age, the general practitioner diagnoses COPD if they have symptoms of dyspnea and / or cough, whether or not they give up with mucus, in combination with a relevant smoking history (> 20 years of smoking or > 15 pack years) and a deviating FEV1 / FVC ratio (< 5th percentile) after standardized bronchodilation	NHG	2015	ME	/
2.4	It is preferable that (ex-) smoking people (over 40 years old) with chronic cough and/or the use of inhalation therapy or more than two lower respiratory tract infections per year are actively screened for COPD diagnosis. This is initially done by means of spirometric research.	LAN	2016	ME	/

2.5	COPD should be considered in all current and former smokers aged > 35 years with symptoms such as breathlessness, cough and sputum production	COPD-X	2017	ME	/
2.6	Patients with any symptoms of COPD (i.e. dyspnoea, chronic cough or chronic sputum production) should undergo spirometry to assess for the presence of COPD	MoHS	2018	ME	Grade D, Level 4
2.7	A diagnosis of COPD should be considered in any patient with a history of exposure to risk factors (e.g., tobacco smoking) and/or with dyspnea (progressive, on exertion or persistent), chronic cough or sputum production	SwRC	2018	ME	/
2.8	People aged over 35 years who present with a risk factor (smoking history, occupational exposure to harmful fumes, dust or chemicals or exposure to fumes, such as biomass fuels) and one or more symptoms (exertional breathlessness, chronic cough, regular sputum production, frequent winter 'bronchitis', or wheeze) of chronic obstructive pulmonary disease (COPD) have post-bronchodilator spirometry.	NICE	2016	ME	/
2.9	Patients who are older than 40 years of age and who are current or ex-smokers should undertake spirometry if they have persistent and progressive exertional dyspnoea, cough, sputum production, wheezing and chest tightness.	MoHS	2018	ME	Grade D, Level 4
2.10	The COPD risk test can be used as a case finding practice. This is a relatively simple method for detecting patients between 40 and 70 years of age with undiagnosed COPD in general practice. For persons who score high risk with the test, further diagnostics are performed, including spirometry. The risk test is particularly distinctive in patients who smoke and cough.	NHG	2015	EX	
2.11	The possibility of COPD should be considered in any smoker with a productive cough.	FiMSD	2019	EX	
2.12	COPD may present as recurrent episodes of chest infection requiring antibiotics	COPD-X	2017	EX	
2.13	All patients ≥ 40 years of age with a history of smoking should be assessed on a yearly basis for symptoms of COPD, i.e. dyspnoea, chronic cough or chronic sputum production	MoHS	2018	EX	
**3**	**Diagnosis**				
3.1	*Medical history interview*				
3.1.1	Smoking is the most important risk factor in COPD development	COPD-X	2017	EX	

3.1.2	Ask the following symptoms that may fit with COPD, physical and social limitations and risk factors for COPD: coughing, wheezing, dyspnea, problems with coughing up mucus, unwanted weight loss or muscle strength loss, influence of the symptoms on daily functioning and at night, influence of the symptoms on the capacity for physical exertion, absenteeism and incapacity for work, current or former smoking behavior, number of smoking years and the average number of cigarettes per day, age when smoking started, working conditions and leisure activities in which one is exposed to gases, vapors, aerosols, dust or smoke, familial occurrence of COPD and drug use	NHG	2015	EX	
3.1.3	Cough and sputum production are the most common symptoms.	FiMSD	2019	EX	
3.1.4	Symptoms can be assessed by the CAT test (COPD Assessment Test™) 2 or mMRC scale (modified Medical Research Council dyspnoea scale)	FiMSD	2019	EX	
3.1.5	A non-smoker may also develop COPD as a result of outdoor or indoor air pollution and passive exposure to smoke.	FiMSD	2019	EX	
3.1.6	Most patients with COPD are smokers.	FiMSD	2019	EX	
3.1.7	Patients with progressive disease suffer from gradually increasing dyspnea on exertion	FiMSD	2019	EX	
3.1.8	The symptoms are usually aggravated in association with respiratory tract infections	FiMSD	2019	EX	
3.1.9	The COPD Assessment Test (CAT) can determine the impact of COPD symptoms on wellbeing and daily life	COPD-X	2017	EX	
3.1.10	The history of moderate and severe exacerbations (including prior hospitalizations) should be recorded	GOLD	2019	EX	
3.1.11	A comprehensive assessment of symptoms is recommended using measures such as the COPD Assessment Test (CAT) and The COPD Control Questionnaire (The CCQ)	GOLD	2019	EX	
3.1.12	COPD symptoms should be quantified using the CAT score upon diagnosis and repeated every 3-6 months during follow-up.	MoHS	2018	EX	

3.1.13	A focused history should be collected in patients with suspected COPD and should include (1) key symptoms such as dyspnea, chronic cough, and sputum production; (2) smoking habits (including quantification of cumulative smoking history, that is, how many packs per year); (3) exposure to other risk factors (i.e., occupational or environmental exposures); (4) past medical history, including asthma and respiratory infections in childhood; (5) family history of COPD or other chronic respiratory disease; (6) pattern of symptom development; (7) history of exacerbations or previous hospitalizations for respiratory disorders; (8) presence of comorbidities; (9) impact of disease on patient's life; (10) opportunities for reducing risk factors (e.g., smoking cessation); and (11) family and social support	SwRC	2018	EX	
3.1.14	The impact of respiratory symptoms on a patient's life should be assessed by the mMRC questionnaire and the CAT or the CCQ	SwRC	2018	EX	
3.1.15	The mMRC dyspnea scale should be used to grade the breathlessness according to the level of exertion required to elicit it	SwRC	2018	EX	
3.2	*Physical examination*				
3.2.1	The general practitioner inspects the patient and pays attention for the degree of dyspnea, the respiratory rate, the use of auxiliary respiratory muscles and inspiration position of the thorax. The general practitioner does an auscultation of the heart and lungs and pays attention for extended expirium, expiratory wheezing and crepitations. If there is severe dyspnea blood pressure, respiratory rate and heart rate has to be determined. Also weight and height has to be measured and compared with previous data.	NHG	2015	EX	
3.2.2	The following symptoms indicate severe COPD (their absence does not exclude mild COPD): Because of airway obstruction, wheezing is heard at the end of forced expiration. A patient with emphysema may have a barrel-chested appearance. On auscultation reduced breath sounds are heard, and on percussion the sound is hyperresonant. There may be cyanosis associated with hypoxaemia.	FiMSD	2019	EX	
3.2.3	Chest auscultation may or may not detect crackles or wheezing with a prolonged expiratory phase	SwRC	2018	EX	
3.2.4	Evidence of fatigue, weight loss and anorexia may be noted in severe cases of COPD	SwRC	2018	EX	
3.2.5	The objective assessment of exercise capacity in patients with limited exercise tolerance is a useful tool in COPD prognosis, in the assessment of health status, as well as, in the assessment of the effectiveness of pulmonary rehabilitation. The paced shuttle walk test and unpaced 6-min walk test are common test modalities.	SwRC	2018	EX	

3.3	*Technical investigations*				
3.3.1	Spirometry is required to make the diagnosis; the presence of a post-bronchodilator FEV1/FVC < 0.70 confirms the presence of persistent airflow limitation.	GOLD<br>COPD-X<br>SwRC<br>FiMSD<br>MoHS	2019<br>2017<br>2018<br>2019<br>2018	IN	/<br>III-2, strong<br>/<br>/<br>Grade D, Level 4
3.3.2	A reduced ratio of forced expiratory volume to forced vital capacity (FEV1/FVC according to the new reference values z < –1.65) in post-bronchodilator spirometry is consistent with COPD.	FiMSD	2019	ME	/
3.3.3	Diagnostic spirometry is performed in all patients with anamnestic indications for COPD. Determine the FEV1, the FVC and a flow volume curve. Airway obstruction: a FEV1/FVC value smaller than the 5th percentile of the reference population ("p5"; also called lower limit of normal 5%, LLN5%) after bronchodilatation.	NHG	2015	ME	/
3.3.4	COPD is excluded with a normal FEV1/FVC ratio (≥ 5th percentile) after standardized bronchodilation	NHG	2015	ME	/
3.3.5	In case of abnormal spirometry, repeat the test after 6 weeks, so that the patient is recovered from a possibly first presented exacerbation of COPD.	NHG	2015	IN	/
3.3.6	Investigations to confirm or exclude other conditions with a similar presentation to COPD (eg, bronchiectasis, lung cancer, heart failure and anaemia) include chest x-ray, haematology and biochemistry, complex lung function tests, exercise stress testing, and electrocardiography and echocardiography	COPD-X	2017	EX	
3.3.7	Single FEV1 meters that cannot produce a flow volume curve are not recommended for diagnostic spirometry	NHG	2015	EX	
3.3.8	Repeat the spirometry after 6 to 12 weeks: if slightly reduced values of the FEV1/FVC ratio after bronchodilation and to evaluate the effect of treatment in case of doubt between asthma and COPD, preferably at the time of symptoms.	NHG	2015	EX	
3.3.9	X-thorax if there is a discrepancy between the symptoms or burden of disease and the spirometric abnormalities (chronic cough or dyspnea and relatively small spirometric abnormalities)	NHG	2015	EX	
3.3.10	High-resolution CT if there is a discrepancy between the symptoms or burden of disease and the spirometric abnormalities (chronic cough or dyspnea and relatively small spirometric abnormalities)	NHG	2015	EX	
3.3.11	Spirometry, before and after bronchodilation, is performed and assessed by trained staff. Conditions for performing spirometry are sufficient training and experience in measuring and interpreting the results and performing periodic check measurements.	LAN	2016	EX	

3.3.12	In addition to medical history interview and physical examination, a condition for making the diagnosis is demonstrate airway obstruction by spirometry.	LAN	2016	EX	
3.3.13	The quality of the medical equipment (including lung function meter and scale) should be checked periodically (annually).	LAN	2016	EX	
3.3.14	If a patient develops COPD before the age of 45 or after an exceptionally short period of smoking (less than 20 pack years), serum alpha1-antitrypsin levels should be determined.	FiMSD	2019	EX	
3.3.15	Pulse oximetry can be used to assess the need for consulting a specialist when considering oxygen therapy.	FiMSD	2019	EX	
3.3.16	HRCT reveals clearly even minor changes associated with emphysema, but this examination is not needed to diagnose COPD.	FiMSD	2019	EX	
3.3.17	In patients requiring admission or when pneumonia is suspected, a chest x-ray should be performed and pneumonia treatment should follow guidelines.	COPD-X	2017	EX	
3.3.18	A chest X-ray should be done when a diagnosis of COPD is suspected.	MoHS	2018	EX	
3.3.19	Personnel conducting spirometry testing should be trained in the conduct of the test and be familiar with the machines they are using	MoHS	2018	EX	
3.3.20	Spirometries should be undertaken when patients are clinically stable and free from respiratory tract infections	MoHS	2018	EX	
3.3.21	Sending sputum samples for culture in primary care is not recommended	MoHS	2018	EX	
3.3.22	In all patients with an exacerbation referred to hospital, a chest radiograph should be obtained and is useful in excluding alternative diagnoses	MoHS	2018	EX	
3.3.23	Measuring arterial blood gas tensions should be considered and the inspired oxygen concentration should be recorded	MoHS	2018	EX	
3.3.24	Theophylline level should be measured in patients on theophylline therapy at admission to rule out toxicity	MoHS	2018	EX	
3.3.25	COPD cannot be diagnosed based on a chest X-ray, but a chest X-ray may be valuable during the initial evaluation to exclude other diseases and to establish the presence of significant comorbidities such as concomitant respiratory, skeletal and cardiac disease.	SwRC	2018	EX	
3.3.26	Consider CT of the chest not only for the differential diagnosis, but also for detection of concomitant bronchiectasis, screening for lung cancer, assessment for lung-volume-reduction procedures and lung transplantation	SwRC	2018	EX	
3.3.27	Measurement of lung volumes by body plethysmography can help characterize COPD severity	SwRC	2018	EX	

3.3.28	Measurement of the diffusing capacity of the lungs for carbon monoxide (DLCO) may be considered to provide an index of the severity of tissue destruction in emphysema	SwRC	2018	EX	
3.3.29	Pulse oximetry is recommended for all patients with clinical signs suggestive of right heart failure or respiratory failure. Pulse oximetry may be useful in identifying possible hypoxic patients who require supplementary oxygen therapy or in determining which patients require arterial blood gas measurements	SwRC	2018	EX	
3.3.30	People with stable chronic obstructive pulmonary disease (COPD) and a persistent resting stable oxygen saturation level of 92% or less have their arterial blood gases measured to assess whether they need long-term oxygen therapy (LTOT)	NICE	2016	EX	
3.3.31	Assessing people for LTOT should comprise measuring arterial blood gases on 2 occasions at least 3 weeks apart in people who have a confident diagnosis of COPD, who are receiving optimum medical management and whose COPD is stable.	NICE	2016	EX	
3.3.32	The WHO recommends that all patients with a diagnosis of COPD should be screened once for alpha-1 antitrypsin deficiency (AATD) especially in areas with high AATD prevalence. A low concentration (< 20% normal) is highly suggestive of homozygous deficiency. Family members should also be screened.	GOLD	2019	EX	
3.3.33	Screening for alpha-1 antitrypsin deficiency is recommended for all patients with diagnosed COPD. A low concentration (< 20% normal) is highly suggestive of homozygous deficiency. Family members should be screened and together with the patient, referred to specialist centres for advice and management	SwRC	2018	EX	
3.3.34	Spirometric tests are not recommended during an exacerbation of COPD	MoHS	2018	EX	
3.3.35	Spirometry should also be performed before a patient is discharged from hospital or at least 4-6 weeks thereafter	SwRC	2018	EX	
3.3.36	Spirometry should be performed after the administration of an adequate dose of at least one short-acting inhaled bronchodilator in order to minimize variability.	GOLD	2019	EX	
3.3.37	For bronchodilation to do a spirometry, use a beta2-sympathomimetic (e.g. salbutamol 400 µg inhalation aerosol).	FiMSD	2019	EX	
**4**	**Treatment**				
4.1	*Prevention and nonpharmacological*				
4.1.1	Smoking cessation is recommended for all patients with COPD	GOLD<br>COPD-X<br>NHG<br>FiMSD<br>MoHS<br>SwRC<br>USPSTF	2019<br>2017<br>2015<br>2019<br>2018<br>2018<br>2016	IN	Evidence A<br>II strong<br>/<br>/<br>Grade A, Level 1<br>Evidence A<br>Grade D

4.1.2	It is recommended that clinicians ask all adults, including pregnant women, about tobacco use	USPSTF	2016	IN	Grade D
4.1.3	A comprehensive approach to supporting smoking cessation involves behavioural support and treatment of nicotine dependence	COPD-X	2017	EX	
4.1.4	Efficient ventilation, non-polluting cooking stoves and similar interventions should be recommended	GOLD	2019	EX	
4.1.5	Clinicians should advise patients to avoid continued exposures to potential irritants, if possible	GOLD	2019	EX	
4.1.6	For patients with COPD, at any stage of the condition, smoking cessation is by far the most effective treatment option to prevent (accelerated) deterioration of lung function and disease progression. The patient receives urgent stop advice. It is pointed out that smoking plays an important role in the development and prognosis of the symptoms and that quitting smoking is an indispensable part of the treatment, just like the use of medicines.	LAN	2016	EX	
4.1.7	It is recommended that clinicians provide interventions, including education or brief counseling, to prevent initiation of tobacco use in school-aged children and adolescents	USPSTF	2016	EX	
4.1.8	Since smoking is the most relevant risk factor for COPD, the indicated prevention of COPD focuses on quitting smoking.	LAN	2016	EX	
4.1.9	Nonpharmacological strategies should be provided to all patients with COPD	COPD-X	2017	EX	
4.1.10	Annual influenza vaccination is recommended for all patients with COPD	GOLD<br>COPD-X<br>NHG<br>LAN<br>FiMSD<br>MoHS<br>SwRC	2019<br>2017<br>2015<br>2016<br>2019<br>2018<br>2018	IN	Evidence B<br>I strong<br>/<br>/<br>/<br>Grade A, level 1<br>Evidence B
4.1.11	Pneumococcal vaccination: the PCV13 and PPSV23 are recommended for all patients ≥ 65 years of age, and in younger patients with significant comorbid conditions including chronic heart or lung disease	GOLD	2019	ME	Evidence B
4.1.12	Patients aged ≥ 50 years who are immunised with polysaccharide pneumococcal vaccine, along with revaccination 5 years later, will have protection against community-acquired pneumonia and a reduced likelihood of COPD exacerbations	COPD-X	2017	ME	I strong
4.1.13	Pneumococcal vaccination (conjugate vaccine) is recommended	FiMSD	2019	ME	/
4.1.14	Pneumococcal vaccination should be considered in COPD patients	MoHS	2018	ME	Grade C, Level 2

4.1.15	A pneumococcal vaccination provides some protection against community-acquired pneumonia (CAP) and is recommended for all COPD patients ≥ 65 years of age. For younger COPD patients, the vaccination is recommended if significant comorbidities, such as chronic heart or lung disease, are present. A single dose of conjugated 13-valent vaccine with no booster doses, is recommended	SwRC	2018	ME	/
4.1.16	No evidence was found for the usefulness of pneumococcal vaccination in COPD.	NHG	2015	EX	
4.1.17	Nutritional supplementation should be considered in malnourished patients with COPD	GOLD<br>FiMSD	2019<br>2019	EX	
4.1.18	The general practitioner advises the patient to exercise sufficiently (for example, half an hour of moderate intensive walking, cycling, swimming or fitness daily)	NHG<br>FiMSD	2015<br>2019	EX	
4.1.19	For all patients with COPD, regardless of the severity of their disease burden, information and education about a healthy lifestyle and dealing with the consequences of the course of the disease are essential. It is recommended to start patient-oriented with information and educational activities as soon as possible after diagnosis. The patient receives step-by-step information about what his illness entails, what treatment is being proposed and what the effects can be on daily life.	LAN	2016	EX	
4.1.20	A COPD patient with a reduced nutritional status is offered, after other causes of this nutritional status have been excluded, a dietary intervention combined with an exercise intervention.	LAN	2016	EX	
4.1.21	All COPD patients receive exercise advice.	LAN	2016	EX	
4.1.22	In the absence of instruction from a specialist exercise professional (eg, physiotherapist or exercise physiologist), individuals with COPD should be encouraged to be physically active	COPD-X	2017	EX	
4.1.23	Participation in activities of daily living that require muscle strength (eg, lifting or squatting for gardening), as well as activities such as bowls, golf, swimming and Tai Chi are recommended. Regular exercise should be provided to all symptomatic COPD patients.	COPD-X	2017	EX	
4.1.24	Physical activity is a strong predictor of mortality. Patients should be encouraged to increase the level of physical activity although we still don't know how to best insure the likelihood of success	GOLD	2019	EX	
4.1.25	Prescribe supplemental oxygen to COPD patients if Pa02 ≤ 55 mmHg or SaO2 < 88% or PaO2 > 55 but < 60 mmHg with right heart failure or erythrocytosis. Titrate to keep SaO2 ≥ 90% and recheck in 60-90 days to assess if supplemental oxygen is still indicated or if prescribed supplemental oxygen is effective.	GOLD	2019	ME	Evidence A
4.1.26	Long-term oxygen therapy is indicated in patients with severe COPD who are in chronic respiratory failure (blood oxygen saturation SpO2 ≤ 88%)	MoHS	2018	ME	Grade A, Level 1

4.1.27	Long-term oxygen therapy (> 16h per day) is recommended to increase the survival in stable COPD patients with arterial hypoxemia (PaO2 ≤ 55 mmHg or SaO2 ≤ 88%, or 55< PaO2 < 60 mmHg or SaO2 = 88%), if there is evidence of pulmonary hypertension, peripheral oedema suggesting congestive cardiac failure, or polycythemia	SwRC	2018	ME	Evidence A
4.1.28	For COPD patients with acute exacerbation, controlled oxygen should be given to keep the blood oxygen saturation within a target saturation of 88-92%	MoHS	2018	EX	
4.1.29	Non-invasive ventilation should be used as the treatment of choice for persistent hypercapnic ventilatory failure during exacerbations of COPD despite optimal medical therapy	MoHS	2018	EX	
4.1.30	When patients are started on non-invasive ventilation, there should be a clear plan covering what to do in the event of deterioration and ceilings of therapy should be agreed	MoHS	2018	EX	
4.1.31	Non-invasive ventilation should be used to facilitate liberation from invasive ventilation in patients recovering from an exacerbation of COPD but who fail spontaneous breathing trials	MoHS	2018	EX	
4.1.32	Non-drug interventions and lifestyle measures such as smoking cessation programs, vaccinations and pulmonary rehabilitation is recommended, and usage of these resources should be maximized in all COPD patients	SwRC	2018	EX	
4.1.33	NIV should be the first mode of ventilation used in COPD patients with acute respiratory failure who have no absolute contraindication because it improves gas exchange, reduces the work of breathing and the need for intubation, decrease hospitalization duration and improves survival	SwRC	2018	EX	
4.1.34	People with an acute exacerbation of chronic obstructive pulmonary disease (COPD) and persistent acidotic hypercapnic ventilatory failure that is not improving after 1 hour of optimal medical therapy have non-invasive ventilation.	NICE	2016	EX	
4.1.35	Non-invasive ventilation should be given once it is recognised that a person is not responding to 1 hour of optimal medical therapy (controlled oxygen therapy, nebulised bronchodilator therapy, systemic corticosteroids and antibiotics if indicated)	NICE	2016	EX	
4.1.36	Consider long-term oxygen therapy for people with COPD who do not smoke and who: have a partial pressure of oxygen in arterial blood (PaO2) below 7.3kPa when stable or have a PaO2 above 7.3 and below 8 kPa when stable, if they also have 1 or more of the following: secondary polycythaemia, peripheral oedema or pulmonary hypertension	NICE	2018	EX	
4.1.37	Advise people who are having long-term oxygen therapy that they should breathe supplemental oxygen for a minimum of 15 hours per day	NICE	2018	EX	

4.1.38	Do not offer long-term oxygen therapy to treat isolated nocturnal hypoxaemia caused by COPD	NICE	2018	EX	
4.1.39	Oxygen therapy at home can be used to prevent elevation of pulmonary arterial pressure in advanced COPD and to prolong survival	FiMSD	2019	EX	
4.1.40	Nocturnal noninvasive ventilation (NIV; with or without oxygen therapy) can be considered if the patient has any of the following during appropriate therapy: hypercapnia (PaCO2 exceeding 7 kPa), corrected by at least 20% with NIV; recurrent exacerbations, during which noninvasive or invasive ventilation is needed, or marked hypercapnia during oxygen therapy, and symptoms of nocturnal hypoventilation.	FiMSD	2019	EX	
4.1.41	If hypoxaemia is present, the SpO2 target range should be 88-92%. This can usually be achieved by the administration of oxygen via nasal cannula at a rate of 0,5-2L/min.	COPD-X	2017	EX	
4.1.42	NIV is effective for patients with rising PaCO2 levels	COPD-X	2017	EX	
4.1.43	In patients with stable COPD and resting or exercise-induced moderate desaturation, long-term oxygen treatment should not be prescribed routinely. However, individual patient factors must be considered when evaluating the patient's need for supplemental oxygen.	GOLD	2019	EX	
4.1.44	In patients with severe chronic hypercapnia and a history of hospitalization for acute respiratory failure, long term noninvasive ventilation may be considered	GOLD	2019	EX	
4.1.45	Titrating oxygen therapy to ≥ 90% saturation is recommended in patients with an AECOPD to avoid hypoxemia and to reduce the risk of oxygen-induced hypercapnia	SwRC	2018	EX	
4.1.46	People receiving emergency oxygen for an acute exacerbation of chronic obstructive pulmonary disease (COPD) have their oxygen saturation levels maintained between 88% and 92%	NICE	2016	EX	
4.1.47	Do not offer long-term oxygen therapy to people who continue to smoke despite being offered smoking cessation advice and treatment, and referral to specialist stop smoking services	NICE	2018	EX	
4.1.48	Do not offer ambulatory oxygen to manage breathlessness in people with COPD who have mild or no hypoxaemia at rest	NICE	2018	EX	
4.1.49	Do not offer short-burst oxygen therapy to manage breathlessness in people with COPD who have mild or no hypoxaemia at rest	NICE	2018	EX	
4.2	*Pharmacological*				
4.2.1	For patients with a mild disease burden, it may also be decided to take 'if necessary' medication, starting with worsening of the symptoms.	NHG	2015	EX	
4.2.2	Pharmacological interventions should always be combined with non-pharmacological interventions.	NHG	2015	EX	

4.2.3	It is recommended that a stepwise approach to pharmacotherapy be used, until adequate control is achieved	COPD-X	2017	EX	
4.2.4	It is recommended that a stepwise approach to pharmacotherapy be used, until adequate control is achieved	COPD-X	2017	EX	
4.2.5	Pharmacological therapy can reduce COPD symptoms, reduce the frequency and severity of exacerbations, and improve health status and exercise tolerance.	GOLD	2019	EX	
4.2.6	Each pharmacological treatment regimen should be individualized and guided by the severity of symptoms, risk of exacerbations, side-effects, comorbidities, drug availability and cost, and the patient's response, preference and ability to use various drug delivery devices	GOLD	2019	EX	
4.2.7	Treatment should be individualized	SwRC	2018	EX	
4.2.8	Group B patients are likely to have comorbidities that may add to their symptomatology and impact their prognosis, and these possibilities should be investigated.	GOLD	2019	EX	
4.2.9	Inhaled bronchodilators are recommended over oral bronchodilators	GOLD<br>SwRC<br>LAN	2019<br>2018<br>2016	ME	Evidence A<br>Evidence A<br>/
4.2.10	Bronchodilators are recommended as the initial treatment for all COPD groups	SwRC	2018	ME	Evidence A
4.2.11	LABAs and LAMAs are preferred over short-acting agents except for patients with only occasional dyspnea, and for immediate relief of symptoms in patients already on long-acting bronchodilators for maintenance therapy	GOLD<br>SwRC	2019<br>2018	ME	Evidence A<br>Evidence A
4.2.12	Short-acting bronchodilators (b2-agonists) are used as needed for short term symptom relief. If these are insufficient, then long-acting bronchodilators should be added.	COPD-X	2017	ME	/
4.2.13	Short-acting bronchodilators are prescribed on an as-needed basis and should be the initial empirical treatment for the relief of breathlessness and exercise limitation.	MoHS	2018	ME	Grade A, Level 1
4.2.14	Rescue short-acting bronchodilators should be prescribed to all patients for immediate symptom relief	GOLD	2019	IN	/
4.2.15	All group A patients should be offered bronchodilator treatment based on its effect on breathlessness. This can be either a short- or a long-acting bronchodilator. This should be continued if benefit is documented.	GOLD	2019	IN	/
4.2.16	For group B, initial therapy should consist of a long acting bronchodilator. Long-acting inhaled bronchodilators are superior to short-acting bronchodilators taken as needed, and are therefore recommended. There is no evidence to recommend one class of long-acting bronchodilators over another for initial relief of symptoms in this group of patients. In the individual patient, the choice should depend on the patient's perception of symptom relief.	GOLD	2019	ME	Evidence A

4.2.17	Patients with persistent breathlessness (GOLD group B) should receive a LABA or a LAMA. If a LAMA is started, SAMA (including nebulisations) should be stopped. Patients with persistent breathlessness should be escalated to a LABA/LAMA combination.	MoHS	2018	ME	Grade A, Level 1
4.2.18	Patients in group B with persistent dyspnea should escalate to two bronchodilators	SwRC	2018	ME	Evidence A
4.2.19	If breathlessness or exacerbations persist with monotherapy, a fixed dose combination LAMA/LABA inhaler is recommended	COPD-X	2017	ME	/
4.2.20	For group C patients, initial therapy should consist of a single long acting bronchodilator. Starting therapy with a LAMA is recommended in this group.	GOLD<br>MoHS	2019<br>2018	IN	/<br>Grade A, Level 1
4.2.21	For patients in group C, a second long-acting bronchodilator is the preferred treatment option if exacerbations persist.	SwRC<br>MoHS	2018<br>2018	IN	Evidence A<br>Grade A, Level 1
4.2.22	Patients with persistent symptoms and frequent exacerbations (GOLD group D) should be started first on a LABA/LAMA combination.	MoHS	2018	ME	Grade A, Level 1
4.2.23	In group D, the decision to use LABA/LAMA as initial treatment should be guided by the level of symptoms.	GOLD	2019	ME	/
4.2.24	For patients with persistent exacerbations on long acting bronchodilator monotherapy, escalation to either LABA/LAMA or LABA/ICS is recommended.	GOLD	2019	ME	/
4.2.25	For patients with frequent exacerbations (more than 2 per year) and persistent breathlessness with FEV1 < 50% of predicted (GOLD group C and D), the use of combination therapy (LABA/ICS or LABA/LAMA) is recommended.	MoHS	2018	ME	Grade A, Level 1
4.2.26	Addition of ICS to standard therapy should be considered for patients with moderate to severe COPD (GOLD group C and D) with frequent exacerbations. The expected benefit of reduction in exacerbations should be balanced against risk of pneumonia.	MoHS	2018	ME	Grade A, Level 1
4.2.27	In patients who develop further exacerbations on LABA/LAMA therapy we suggest two alternative pathways: escalation to LABA/LAMA/ICS if blood eosinophil counts ≥ 100 cells/microliter or add roflumilast or azithromycin if blood eosinophil < 100 cells/microliter	GOLD	2019	IN	/
4.2.28	In patients who develop further exacerbations on LABA/ICS therapy, we recommend escalation to triple therapy by adding a LAMA.	GOLD	2019	IN	/
4.2.29	For patients with COPD and few symptoms (for example MRC < 2 or CCQ < 1), inhalation medication may not be required	NHG	2015	EX	
4.2.30	Start with one of the two types of short-acting bronchodilators: a SABA or a SAMA	NHG	2015	EX	
4.2.31	Choose the other type of bronchodilator if there is insufficient improvement (persistent symptoms of dyspnea) after two weeks or add an agent of the other type	NHG	2015	EX	

4.2.32	If patients do not achieve their treatment goals with (moderate) severe airway obstruction (FEV1 < 80% of predict) switch to maintenance treatment with a long-acting bronchodilator: a LABA or LAMA	NHG	2015	EX	
4.2.33	Consider adding inhaled corticosteroids (ICS) for one year only to patients with frequent severe exacerbations (two or more treatments with prednisolon or an antibiotic or hospitalization for COPD per year), despite maintenance treatment with a long-acting bronchodilator	NHG	2015	EX	
4.2.34	If the number of exacerbations does not clearly decrease after one year or if there are no more exacerbations for a longer period (two years), treatment with ICS will be discontinued	NHG	2015	EX	
4.2.35	The general practitioner does not generally start maintenance treatment with a combination preparation of an ICS and a LABA, due to the limited indication of ICS in COPD	NHG	2015	EX	
4.2.36	Bronchodilator medications in COPD are central to symptom management and commonly given on a regular basis to prevent or reduce symptoms.	GOLD	2019	EX	
4.2.37	Maintenance therapy with long-acting bronchodilators should be initiated as soon as possible before hospital discharge	GOLD	2019	EX	
4.2.38	Use of short acting bronchodilators on a regular basis is not generally recommended.	GOLD	2019	EX	
4.2.39	Patients may be started on single long-acting bronchodilator therapy or dual long-acting bronchodilator therapy. In patients with persistent dyspnea on one bronchodilator treatment should be escalated to two.	GOLD	2019	EX	
4.2.40	For patients in group B with severe breathlessness initial therapy with two bronchodilators may be considered.	GOLD	2019	EX	
4.2.41	For patients in group C, LABA/ICS can be considered if exacerbations persist.	SwRC<br>MoHS	2018<br>2018	EX	
4.2.42	For group D in general, therapy can be started with a LAMA as it has effects on both breathlessness and exacerbations. For patients with more severe symptoms (order of magnitude ≥ 20), especially driven by greater dyspnea and/or exercise limitation, LAMA/LABA may be chosen as initial treatment. The decision to use LABA/LAMA as initial treatment should be guided by the level of symptoms.	GOLD	2019	EX	
4.2.43	In some patients of group D, initial therapy with LABA/ICS may be the first choice. This treatment has the greatest likelihood of reducing exacerbations in patients with blood eosinophil counts ≥ 300 cells/microliter. LABA/ICS may also be first choice in COPD patients with a history of asthma.	GOLD	2019	EX	
4.2.44	Combination treatment with a LABA/LAMA reduces exacerbations compared to monotherapy	GOLD	2019	EX	

4.2.45	Combination treatment with a LABA and LAMA increases FEV1 and reduces symptoms compared to monotherapy	GOLD	2019	EX	
4.2.46	LAMAs have a greater effect on exacerbation reduction compared with LABAs and decrease hospitalizations	GOLD	2019	EX	
4.2.47	LABAs and LAMAs significantly improve lung function, dyspnea, health status, and reduce exacerbation rates	GOLD	2019	EX	
4.2.48	Regular and as-needed use of SABA or SAMA improves FEV1 and symptoms	GOLD	2019	EX	
4.2.49	Combinations of SABA and SAMA are superior compared to either medication alone in improving FEV1 and symptoms	GOLD	2019	EX	
4.2.50	Offer LAMA + LABA to people who have spirometrically confirmed COPD and do not have asthmatic features/features suggesting steroid responsiveness and remain breathless or have exacerbations despite: having used or been offered treatment for tobacco dependence if they smoke and optimised non-pharmacological management and relevant vaccinations and using a short-acting bronchodilator	NICE	2018	EX	
4.2.51	Consider LABA + ICS for people who have spirometrically confirmed COPD and have asthmatic features/features suggesting steroid responsiveness and remain breathless or have exacerbations despite: having used or been offered treatment for tobacco dependence if they smoke and optimised non-pharmacological management and relevant vaccinations and using a short-acting bronchodilator	NICE	2018	EX	
4.2.52	Before starting LAMA + LABA + ICS, conduct a clinical review to ensure that the person's non-pharmacological COPD management is optimised and they have used or been offered treatment for tobacco dependence if they smoke, acute episodes of worsening symptoms are caused by COPD exacerbations and not by another physical or mental health condition, the person's day-to-day symptoms that are adversely impacting their quality of life care caused by COPD and not by another physical or mental health condition	NICE	2019	EX	
4.2.53	For people with COPD who are taking LABA + ICS, offer LABA + LAMA + ICS if: their day-to-day symptoms continue to adversely impact their quality of life or they have a severe exacerbation (requiring hospitalisation) or they have 2 moderate exacerbations within a year	NICE	2019	EX	
4.2.54	For people with COPD who are taking LAMA + LABA, consider LABA + LAMA + ICS if: they have a severe exacerbation (requiring hospitalisation) or they have 2 moderate exacerbations within a year	NICE	2019	EX	

4.2.55	For people with COPD who are taking LAMA + LABA and whose day-to-day symptoms adversely impact their quality of life: consider a trial of LAMA + LABA + ICS, lasting for 3 months only; after 3 months, conduct a clinical review to establish whether or not LAMA + LABA + ICS has improved their symptoms: if symptoms have not improved, stop LAMA + LABA + ICS and switch back to LAMA + LABA, if symptoms have improved, continue with LAMA + LABA + ICS	NICE	2019	EX	
4.2.56	An inhaled corticosteroid/LABA (ICS/LABA) combination inhaler may be considered in cases of more severe COPD (FEV1 < 50% predicted, with a history of repeated exacerbations), although ICS may increase the risk of pneumonia.	COPD-X	2017	EX	
4.2.57	While combination LAMA/LABA inhalers appear to be more beneficial than ICS/LABA inhalers in reducing exacerbations, the use of an ICS/LABA inhaler together with a LAMA inhaler remains an option for patients with moderate to severe COPD who require additional treatment.	COPD-X	2017	EX	
4.2.58	For group D, treatment can be escalated to triple inhaled therapy (ICS/LAMA/LABA)	SwRC	2018	EX	
4.2.59	Document the reason for continuing ICS use in clinical records and review at least annually	NICE	2019	EX	
4.2.60	Long-term treatment with ICS may be considered in association with LABAs for patients with a history of exacerbations despite appropriate treatment with long-acting bronchodilators	GOLD	2019	EX	
4.2.61	ICS may cause side effects such as pneumonia, so should be used as initial therapy only after the possible clinical benefits versus risks have been considered.	GOLD	2019	EX	
4.2.62	If patients treated with LABA/LAMA/ICS who still have exacerbations the following options may be considered: add roflumilast, add a macrolide or stopping ICS	GOLD	2019	EX	
4.2.63	For patients with persistent breathlessness or exercise limitation on long acting bronchodilator monotherapy, the use of two bronchodilators is recommended. Switching inhaler device or molecules can also be considered.	GOLD	2019	EX	
4.2.64	Low risk of exacerbations (FEV1 ≥ 50%, in the preceding year 0 to 1 exacerbations, no exacerbation requiring hospital treatment, and the patient does not show the COPD-asthma phenotype): Patients with few symptoms should primarily be given a short-acting beta2-sympathomimetic with bronchodilating effect, an anticholinergic drug or a combination of the two. If a short-acting bronchodilator is insufficient to alleviate the symptoms or there are abundant symptoms, a long-acting bronchodilating beta2-sympathomimetic or anticholinergic drug or their combination can be tried. These can be combined with theophylline but there is little evidence for its efficacy, and it has significant adverse effects.	FiMSD	2019	EX	

4.2.65	High risk of exacerbations (FEV1 < 50% or at least 2 exacerbations or an exacerbation requiring hospital treatment in the preceding year): First-choice treatment: fixed combination of inhaled glucocorticoid and long-acting beta2-sympathomimetic, or a long-acting anticholinergic drug; Alternative treatments: combination of two long-acting drugs with different bronchodilating action (beta2-sympathomimetic + anticholinergic drug) or roflumilast can be added to other medication (at least to long-acting bronchodilating drugs) if the patient also has chronic bronchitis, poor lung function (FEV1 < 50%) and recurrent exacerbations or triple medication, i.e. an inhaled glucocorticoid, a long-acting beta2-sympathomimetic and a long-acting anticholinergic drug or theophylline can be combined with an inhaled glucocorticoid and/or long-acting bronchodilating drugs.	FiMSD	2019	EX	
4.2.66	A fixed-dose combination of any LABA and LAMA should be considered above monotherapy	SwRC	2018	EX	
4.2.67	The once-daily combination of LAMA/LABA should be considered for maintenance therapy in patients with moderate to severe COPD	SwRC	2018	EX	
4.2.68	Triple therapy should be considered in selected, symptomatic patients with severe airflow obstruction and at least one exacerbation in the previous year	SwRC	2018	EX	
4.2.69	Long-term monotherapy with ICS is not recommended	GOLD<br>SwRC<br>MoHS	2019<br>2018<br>2018	EX	
4.2.70	In patients who develop further exacerbations on LABA/ICS therapy, treatment can be switched to LABA/LAMA if there has been a lack of response to ICS treatment, or if ICS side effects warrant discontinuation	GOLD	2019	EX	
4.2.71	Low-dose long acting oral and parenteral opioids may be considered for treating dyspnea in COPD patients with severe disease	GOLD	2019	EX	
4.2.72	Opioids (oral or parenteral) are effective therapy for the management of refractory dyspnoea and should be considered on an individual basis	MoHS	2018	EX	
4.2.73	Long-term therapy with oral corticosteroids is not recommended	GOLD<br>SwRC	2019<br>2018	EX	
4.2.74	Long-term therapy with oral corticosteroids is reserved for the pulmonologist because of the long-term effects	NHG	2015	EX	
4.2.75	Long-term oral steroids are discouraged in view of unfavourable risk-benefit ratio.	MoHS	2018	EX	
4.2.76	Before starting azithromycin, ensure the person has had: an electrocardiogram (ECG) to rule out prolonged QT interval and baseline liver function tests	NICE	2018	IN	/
4.2.77	Review prophylactic azithromycin after the first 3 months, and then at least every 6 months	NICE	2018	IN	/

4.2.78	Before starting prophylactic antibiotic therapy in a person with COPD, think about whether respiratory specialist input is needed	NICE	2018	EX	
4.2.79	Consider azithromycin (usually 250 mg 3 times a week) for people with COPD if they: do not smoke and have optimised non-pharmacological management and inhaled therapies, relevant vaccinations and (if appropriate) have been referred for pulmonary rehabilitation and continue to have 1 or more of the following, particularly if they have significant daily sputum production: frequent (typically 4 or more per year) exacerbations with sputum production, prolonged exacerbations with sputum production, exacerbations resulting in hospitalisation	NICE	2018	EX	
4.2.80	Before offering prophylactic antibiotics, ensure that the person has had: sputum culture and sensitivity (including tuberculosis culture), to identify other possible causes of persistent or recurrent infection that may need specific treatment (for example, antibiotic-resistant organisms, atypical mycobacteria or Pseudomonas aeruginosa), training in airway clearance techniques to optimise sputum clearance, a CT scan of the thorax to rule out bronchiectasis and other lung pathologies	NICE	2018	EX	
4.2.81	When prescribing azithromycin, advise people about the small risk of hearing loss and tinnitus, and tell them to contact a healthcare professional if this occurs	NICE	2018	EX	
4.2.82	Only continue treatment if the continued benefits outweigh the risks. Be aware that there are no long-term studies on the use of prophylactic antibiotics in people with COPD	NICE	2018	EX	
4.2.83	For people who are taking prophylactic azithromycin and are still at risk of exacerbations, provide a non-macrolide antibiotic to keep at home as part of their exacerbation action plan	NICE	2018	EX	
4.2.84	Be aware that it is not necessary to stop prophylactic azithromycin during an acute exacerbation of COPD	NICE	2018	EX	
4.2.85	Continuous use of macrolides will reduce exacerbations of COPD but their extensive use is restrained by the fear of increased bacterial resistance to macrolides.	FiMSD	2019	EX	
4.2.86	Long-term macrolide treatment (6-12 months) may be considered in a select group of patients who have multiple exacerbations which are refractory to standard therapy. There is insufficient data to recommend routine use of macrolides in the treatment of COPD.	MoHS	2018	EX	
4.2.87	In former smokers with exacerbations despite appropriate therapy, macrolides, in particular azithromycin, can be considered	GOLD	2019	EX	

4.2.88	Although prophylactic antibiotics are not recommended for routine use in patients with COPD, they may be considered in certain situations. Azithromycin 500mg 3 times a week might be considered in patients with severe airflow obstruction and recurrent, frequent exacerbations	SwRC	2018	EX	
4.2.89	Treatment with a macrolide antibiotic (azithromycin 3x per week) can be considered in former smokers, particularly in frequent exacerbators and those requiring long-term oxygen therapy and PDE-4 inhibitors (roflumilast) in patients with an FEV1 < 50% predicated and chronic bronchitis	SwRC	2018	EX	
4.2.90	If the patient exhibits two of the three symptoms (increased dyspnoea, increased sputum or purulent sputum), antimicrobial medication is usually indicated. Options for antimicrobial treatment include amoxicillin, amoxicillin-clavulanic acid, doxycycline and sulpha-trimethoprim, and in recurrent exacerbations moxifloxacin can be considered.	FiMSD	2019	EX	
4.2.91	Theophylline is not recommended unless other long-term treatment bronchodilators are unavailable or unaffordable. Theophylline exerts a small bronchodilator effect in stable COPD and that is associated with modest symptomatic benefits	GOLD<br>SwRC	2019<br>2018	IN	Evidence B<br>Evidence B
4.2.92	Low-dose theophylline may be considered in patients with COPD where symptom control is still not achieved with existing inhaled bronchodilator therapy.	MoHS	2018	EX	
4.2.93	In patients with exacerbations despite LABA/ICS or LABA/LAMA/ICS, chronic bronchitis and severe to very severe airflow obstruction, the addition of a PDE4 inhibitor can be considered	GOLD	2019	EX	
4.2.94	Addition of roflumilast to inhaled bronchodilator therapy may provide benefits in reducing exacerbations in patients with FEV1 < 50% and chronic bronchitis who have recurrent exacerbations despite triple inhaler therapy. However, this must be weighed in the context of increased risk of adverse events.	MoHS	2018	EX	
4.2.95	Mucolytic drug therapy should be considered in patients with a chronic cough productive of sputum and should be continued if there is symptomatic improvement.	MoHS	2018	IN	Grade B, Level 1
4.2.96	Antioxidant mucolytics are recommended only in selected patients	GOLD<br>SwRC	2019<br>2018	EX	
4.2.97	Mucolytic agents may reduce exacerbations of COPD without significant adverse effects but will not improve lung function.	FiMSD	2019	EX	
4.2.98	Acetyl cysteine is not recommended	NHG	2015	EX	
4.2.99	Antitussives cannot be recommended	GOLD	2019	EX	
4.2.100	Statin therapy is not recommended for prevention of exacerbations	GOLD	2019	EX	

4.2.101	Drugs approved for primary pulmonary hypertension are not recommended for patients with a pulmonary hypertension secondary to COPD	GOLD	2019	EX	
4.2.102	Patients with severe hereditary alpha-1 antitrypsin deficiency and established emphysema may be candidates for alpha-1 antitrypsin augmentation therapy	GOLD<br>SwRC	2019<br>2018	EX	
4.2.103	It is suggested that alpha-1 antitrypsin augmentation therapy may be considered for never-smokers or ex-smokers with an FEV1 of 35-60% predicted.	SwRC	2018	EX	
4.2.104	Alpha-1 antitrypsin augmentation therapy may be considered for patients with severe hereditary AATD and established emphysema	SwRC	2018	EX	
4.2.105	For smoking cessation, nicotine replacement therapy is effective in supporting smoking cessation attempts and should be prescribed in the absence of contraindications	SwRC	2018	EX	
4.2.106	Short-acting inhaled beta2-agonists, with or without short-acting anticholinergics, are recommended as the initial bronchodilators to treat an acute exacerbation	GOLD<br>SwRC<br>MoHS	2019<br>2018<br>2018	ME	Evidence C<br>Evidence C<br>Grade C, Level 2
4.2.107	If there is an exacerbation, increased doses of salbutamol, 4-8 puffs, via a metered dose inhaler and spacer every 3-4 hours, should be used.	COPD-X	2017	ME	I strong
4.2.108	Methylxanthines are not recommended for treatment of AECOPD	GOLD<br>SwRC	2019<br>2018	IN	Evidence B<br>Evidence B
4.2.109	Offer 30 mg oral prednisolone daily for 5 days to manage an exacerbation of COPD	NICE	2018	ME	/
4.2.110	In the absence of significant contraindications, oral corticosteroids should be considered in patients in the community who have an exacerbation with a significant increase in breathlessness which interferes with daily activities	MoHS	2018	ME	Grade A, Level 1
4.2.111	A morning dose of oral prednisolone 30-50 mg should be taken for 5 days; tapering the dose is rarely necessary.	COPD-X	2017	ME	I strong
4.2.112	Glucocorticosteroid treatment should not be given for more than 5-7 days	SwRC	2018	ME	Evidence A
4.2.113	For COPD patients with acute exacerbation, prednisolone 30 mg orally should be administered for 5-10 days	MoHS	2018	ME	Grade A, Level 1
4.2.114	Exacerbations with signs and symptoms of infection (increased volume and change in colour of sputum or fever) benefit from antibiotics. First-line agents include oral amoxicillin or doxycycline for 5 days.	COPD-X	2017	ME	II strong
4.2.115	Antibiotics should be used to treat exacerbations of COPD associated with a history of more purulent sputum	MoHS	2018	ME	Grade A, Level 1
4.2.116	The length of antibiotic therapy need not exceed five days for mild to moderate exacerbations of COPD	MoHS	2018	ME	Grade A, Level 1
4.2.117	For moderate to severe exacerbations of COPD, a 7-10 day course of antibiotics is recommended	MoHS	2018	ME	Grade A, Level 1

4.2.118	AECOPDs should not be treated with antibiotics, unless a bacterial infection is suspected, or the patient requires treatment in the intensive care unit. Antibiotic treatment should not exceed 5-7 days during an AECOPD.	SwRC	2018	ME	Evidence B
4.2.119	Treatment of an acute exacerbation: oxygen by nasal catheter or by venturi mask; bi-level positive pressure ventilation improves recovery from severe acute exacerbation of COPD; inhaled beta2-sympathomimetic; the patient's condition permitting, a course of oral glucocorticoids should be started (recommended dose is 30-40 mg prednisolone/day for 5 days)	FiMSD	2019	EX	
4.2.120	In the case of a non-serious exacerbation, the general practitioner recommends: the use of short- and / or long-acting bronchodilators in the maximum dose; if the effect is insufficient, supplemented with a cure of prednisolone 1 dd 40 mg for 5 days or 1 dd 30 mg for 7 days (depending on control after 2 to 5 days extend to a maximum of 14 days); check if necessary	NHG	2015	EX	
4.2.121	Systemic corticosteroids should be used in conjunction with other therapies in all patients admitted to hospital with an exacerbation of COPD	MoHS	2018	EX	
4.2.122	Antibiotics should be given in exacerbations of COPD if the patient requires mechanical ventilation (invasive or non-invasive)	MoHS	2018	EX	
4.2.123	Systemic corticosteroids can improve lung function (FEV1), oxygenation and shorten recovery time and hospitalizaion duration. Duration of therapy should not be more than 5-7 days.	GOLD	2019	EX	
4.2.124	Antibiotics, when indicated, can shorten recovery time, reduce the risk of early relapse, treatment failure, and hospitalization duration. Duration of therapy should be 5-7 days.	GOLD	2019	EX	
4.2.125	At initiation of therapy, education involving instruction, visual demonstration and patient observation is recommended	COPD-X	2017	EX	
4.2.126	Inhaler technique needs to be assessed regularly	GOLD<br>COPD-X	2019<br>2017	EX	
4.2.127	The choice of inhaler device has to be individually tailored and will depend on access, cost, prescriber, and most importantly, patient's ability and preference	GOLD<br>NHG	2019<br>2015	EX	
4.2.128	Base the choice of drugs and inhalers on: how much they improve symptoms, the person's preferences and ability to use the inhalers, the drugs' potential to reduce exacerbations, their side effects and their cost. Minimise the number of inhalers and the number of different types of inhaler used by each person as far as possible	NICE	2018	EX	

4.2.129	It is essential to provide instructions and to demonstrate the proper inhalation technique when prescribing a device, to ensure that inhaler technique is adequate and re-check at each visit that patients continue to use their inhaler correctly	GOLD	2019	EX	
4.2.130	Inhaler technique (and adherence to therapy) should be assessed before concluding that the current therapy requires modification	GOLD	2019	EX	
4.2.131	When prescribing long-acting drugs, ensure people receive inhalers they have been trained to use (for example, by specifying the brand and inhaler in prescriptions)	NICE	2018	EX	
4.2.132	People with chronic obstructive pulmonary disease (COPD) who are prescribed an inhaler have their inhaler technique assessed when starting treatment and then regularly during treatment.	NICE	2016	EX	
4.2.133	Preference is given, if possible, to an inhalant with a dose counter or indicator to reduce the risk of the patient using an empty inhaler. It is recommended that the general practitioner gains experience with a limited number of inhalers.	NHG	2015	EX	
4.2.134	Always ask the patient to take the medication and inhalers with him and to demonstrate inhalation of the medication to correct any errors in the inhalation technique. The inhalation instruction is also a task of the pharmacist. It is recommended to coordinate the instructions.	NHG	2015	EX	
4.2.135	If for the same active substance there is a choice between a powder inhaler single dose and a powder inhaler multidose, then it is generally advisable to prescribe a powder inhaler multidose for ease of use.	NHG	2015	EX	
4.2.136	A medication assessment takes place at least annually. The patient will be instructed by the healthcare provider on how to prepare the inhaler for use, how to administer the dose, how best to store and maintain the device and/or how to check that it is still suitable for use. During the evaluation, the patient shows how he inhales.	LAN	2016	EX	
4.2.137	If the patient uses different types of medication, uniformity is sought in inhalation devices	LAN	2016	EX	
4.2.138	For patients with severe exacerbations that require hospitalization, treatment by vaporization is an option. If a patient makes use of vaporization, the choice of the equipment, its correct use, cleaning and evaluating the result of the treatment is very important. One of the caregivers supports the patient with the proper use and maintenance of the nebulizer and also pays attention to the unwanted inhalation exposure of the patient's housemates to the medication.	LAN	2016	EX	
4.2.139	The treatment choice should consider the patient’s ability to use the device proficiently	COPD-X	2017	EX	

4.2.140	Both nebulisers and hand-held inhalers can be used to administer inhaled therapy during exacerbations of COPD	MoHS	2018	EX	
4.2.141	Patients should be changed to hand-held inhalers as soon as their condition has stabilised	MoHS	2018	EX	
4.2.142	If a patient is hypercapnic or acidotic, the nebuliser should be driven by compressed air, not oxygen (to avoid worsening hypercapnia). If oxygen therapy is needed, it should be administered simultaneously by nasal cannulae	MoHS	2018	EX	
4.2.143	Combination of ICS and LABA in one inhaler should be considered for patients in whom both ICS and LABA are indicated.	MoHS	2018	EX	
**5**	**Management**				
5.1	*Stable COPD*				
5.1.1	The management strategy for stable COPD should be predominantly based on the individualized assessment of symptoms and future risk of exacerbations	GOLD	2019	EX	
5.1.2	Management strategies are not limited to pharmacological treatments and should be complemented by appropriate non-pharmacological interventions	GOLD	2019	EX	
5.1.3	Following implementation of therapy, patients should be reassessed for attainment of treatment goals and identification of any barriers for successful treatment. Following review of the patient response to treatment initiation, adjustments in pharmacological treatment may be needed.	GOLD	2019	EX	
5.1.4	In the case of a 'new' patient with COPD, the general practitioner will check after starting or after a change of pharmacological therapy whether the patient experiences sufficient improvement.	NHG	2015	EX	
5.1.5	The monitoring consists of: determining the burden of disease (evaluation of the symptoms, the limitations experienced, the exercise capacity, number, severity and duration of the exacerbations, FEV1 and nutritional status and the effect of the treatment set on these parameters, using the CCQ or MRC; attention to self-management; discussing problems with smoking cessation and, if necessary, offering extra guidance; attention to adequate exercise, adequate nutrition and optimization of body weight; monitoring compliance and inhalation technique; patient inspection, determination of the weight and possibly auscultation of the lungs; spirometry if necessary	NHG	2015	EX	

5.1.6	In patients who are persistently symptomatic or who have recurrent exacerbations, care should be taken to evaluate compliance, check inhaler technique and exclude other concomitant pathology and comorbidity (e.g. a COPD patient may also be suffering from ischaemic heart disease and congestive cardiac failure, or may have lung cancer or tuberculosis). If these are excluded, treatment of the COPD needs to be optimised.	MoHS	2018	EX	
5.1.7	When assessing whether home treatment is possible, the general practitioner takes into account risk factors for a serious course (age > 65 years, respiratory rate ≥ 30 / min, systolic blood pressure < 90 mmHg, disorientation, renal impairment)	NHG	2015	EX	
5.2	*Exacerbations*				
5.2.1	Following an exacerbation, appropriate measures for exacerbation prevention should be initiated.	GOLD	2019	EX	
5.2.2	In an exacerbation, the general practitioner inspects the patient and auscultates the lungs; traces the cause of the exacerbation; assesses whether there is another (cardiac) cause of worsening dyspnea; measure if doubt about the severity of the exacerbation and the management to be followed (refer or not) the oxygen saturation with a pulse oximeter and perform a CRP determination in patients with a light or moderate airway obstruction and doubt about the severity of the exacerbation	NHG	2015	EX	
5.2.3	In the event of an exacerbation with one or more alarm symptoms, the GP will call an ambulance and in the meantime act according to the following recommendations: If oxygen is present: start with 10 to 15 liters of O2 / min and reduce as quickly as possible on the basis of peripheral oxygen saturation; strive for an oxygen saturation between 90 and 92%; combine this, if nebulizing equipment is available, with nebulisation of salbutamol (2.5 to 5.0 mg) and ipratropium bromide (0.5 mg). If nebulizing equipment is not available: give salbutamol and ipratropium per dose aerosol. In both situations, consider one-time 30 mg prednisolone per os or in patients who are too anxious to swallow dexamethasone 8 mg IM.	NHG	2015	EX	
5.2.4	In the event of a serious exacerbation, wait for the effect of the airway wideners on site; in less serious cases a check can be arranged within a few hours.	NHG	2015	EX	

5.2.5	If improvement: give an oral course of prednisolone (in patients with diabetes mellitus or who have symptoms that are compatible with diabetes mellitus, the glucose value is determined once during the prednisolon course); instructions for the use of bronchodilators during the next 24 hours (for example, a double dose or inhalation through a chamber); check whether there is an indication for antimicrobial agents	NHG	2015	EX	
5.2.6	Initial examinations for an acute exacerbation: status (respiratory rate, skin tone, use of auxiliary breathing muscles, peripheral oedema, auscultation of the heart and lungs, blood pressure), oxygen saturation, chest X-ray and basic blood count	FiMSD	2019	EX	
5.2.7	Early diagnosis and treatment of exacerbations may prevent hospital admission and delay COPD progression	COPD-X	2017	EX	
5.2.8	Exacerbations should be managed promptly with bronchodilators, corticosteroids and antibiotics as appropriate to prevent hospital admission and delay COPD progression	COPD-X	2017	EX	
5.3	*Education and self-management*				
5.3.1	The general practitioner gives lifestyle advice. The most important measures are smoking cessation, adequate exercise and adequate nutrition.	NHG	2015	EX	
5.3.2	Education is needed to change patient's knowledge but there is no evidence that used alone it will change patient behavior	GOLD	2019	EX	
5.3.3	Education self-management with the support of a case manager with or without the use of a written action plan is recommended for the prevention of exacerbation complications such as hospital admissions	GOLD	2019	EX	
5.3.4	The general practitioner encourages the patient to make independent choices with regard to daily activities that may be influenced by COPD and promotes that the patient actively participates in the treatment of the disorder. Important elements in supporting self-management are patient assessment, shared decision making about the approach, a coaching role of the general practitioner and an individual care plan. Self-management appears to be particularly useful in relatively young patients and in patients with a moderately severe disease burden or comorbidity.	NHG	2015	EX	

5.3.5	Important points for attention in self-management are: a healthy lifestyle (no smoking, sufficient exercise, adequate nutrition, avoiding exposure to particulate matter as much as possible); dealing with symptoms and physical, social and psychological consequences of the disorder; adherence to therapy, adequate use of (inhalation) medication, adjusting the dose of bronchodilators in the event of an exacerbation; mobilizing and maintaining social contacts and support	NHG	2015	EX	
5.3.6	With frequent exacerbations (at least two courses of prednisolone or antibiotic or hospital admissions in connection with COPD per year) and highly adequate coping, the patient can in principle start taking medication himself if the symptoms worsen. A condition for this is that the patient is able to recognize the symptoms and knows when he or she should contact if the self-initiated treatment does not work. The general practitioner estimates the possibilities for this, based on the degree of self-management, the burden of disease and the degree of informal care.	NHG	2015	EX	
5.3.7	After discharge, patients should receive self-management education.	COPD-X	2017	EX	
5.4	*Multidisciplinary team management*				
5.4.1	For patients with a mild burden of disease or during the initial phase of COPD, recently diagnosed by the general practitioner, the general practitioner is the main practitioner. For patients with a persistent moderate or severe burden of disease, the pulmonologist is usually the main practitioner.	NHG	2015	EX	
5.4.2	The general practitioner and pulmonologist inform each other about a major change in medication (such as structural change of maintenance medication), preferably digitally and if this is not posible in writing	NHG	2015	EX	
5.4.3	The pulmonologist reports after the diagnosis or a (definitive) treatment plan, upon referral or otherwise at least annually, and meanwhile with new points of view that are important for the general practitioner	NHG	2015	EX	
5.4.4	The general practitioner advises the patient to contact the medical officer if there are work-related problems or obstacles, after which, if necessary, coordination about management is held. The pulmonologist can also play a role in this.	NHG	2015	EX	
5.4.5	With complex somatic and/or adaptation problems the possibility exists of intensive integral analysis by a specialized multidisciplinary team.	LAN	2016	EX	

5.4.6	For a good course of communication and collaboration, there must be a multidisciplinary and up-to-date nursing record that is also available to the (family of the) patient at all times.	LAN	2016	EX	
5.4.7	For COPD patients, with a mild to moderate airway obstruction and a mild to moderate burden of disease, the general practitioner is the main practitioner. He is ultimately responsible for diagnosis and treatment and/or supervision of a patient and is responsible for the continuity of care. The general practitioner is as the main practitioner part of a multidisciplinary team and may consist of: practice nurse/supporter, primary care lung nurse, pharmacist, pharmacist's assistant, psychologist, physical therapist and other (para)medical care providers.	LAN	2016	EX	
5.4.8	For COPD patients with a moderate or severe burden of disease, who receive more specialized treatment, it is obvious that the pulmonologist is the main treatment provider.	LAN	2016	EX	
5.4.9	It is recommended that patients with COPD use one pharmacy in order for the pharmacist to fulfill its medication monitoring task	LAN	2016	EX	
5.4.10	The discharge plan should be promptly shared with the primary care team.	COPD-X	2017	EX	
5.4.11	Clinical support teams working with the primary health care team can help enhance quality of life and reduce disability for patients with COPD	COPD-X	2017	EX	
5.4.12	Multidisciplinary care may assist home management of some patients with an exacerbation	COPD-X	2017	EX	
5.4.13	A plan of care should be developed with the multidisciplinary team. COPD action plans reduce hospitalisations and are recommended as part of COPD self-management.	COPD-X	2017	EX	
**6**	**Comorbidities**				
6.1	Do not offer the following treatments solely to manage pulmonary hypertension caused by COPD, except as part of a randomised controlled trial: bosentan, losartan, nifedipine, nitric oxide, pentoxifylline, phosphodiesterase-5 inhibitors or statins	NICE	2018	EX	
6.2	Ensure that people with cor pulmonale caused by COPD are offered optimal COPD treatment, including advice and interventions to help them stop smoking.	NICE	2018	EX	
6.3	Do not use the following to treat cor pulmonale caused by COPD: alpha-blockers, angiotensin-converting enzyme inhibitors, calcium channel blockers or digoxin (unless there is atrial fibrillation)	NICE	2018	EX	
6.4	COPD often coexists with other diseases (comorbidities) that may have a significant impact on disease course	GOLD	2019	EX	

6.5	In general, the presence of comorbidities should not alter COPD treatment and comorbidities should be treated per usual standards regardless of the presence of COPD	GOLD	2019	EX	
6.6	When COPD is part of a multimorbidity care plan, attention should be directed to ensure simplicity of treatment and to minimize polypharmacy	GOLD	2019	EX	
6.7	Concomitant chronic diseases occur frequently in COPD patients, including cardiovascular disease, skeletal muscle dysfunction, metabolic syndrome, osteoporosis, depression, anxiety, and lung cancer. These comorbidities should be actively sought and treated appropriately when present as they can influence mortality and hospitalizations independently.	GOLD	2019	EX	
6.8	It is recommended that COPD patients are checked annually for cardiovascular diseases and diabetes mellitus	NHG	2015	EX	
6.9	The general practitioner pays attention to psychosocial factors, such as fear of dyspnea, feelings of shame, sexual problems and social isolation, and to symptoms related to anxiety and depression	NHG	2015	EX	
6.10	In patients who are expected to be treated with prednisolone for 3 months or longer at a dose of $\geq$ 15 mg / day, there is an indication, regardless of age, for maintenance treatment with bisphosphonate and vitamin D supplementation, supplemented with calcium supplementation, depending on the calcium intake. This is part of fracture prevention.	NHG	2015	EX	
6.11	It is important that all COPD patients are examined to see if there is comorbidity in the form of an anxiety disorder and / or a depressive disorder, delirium or insomnia.	LAN	2016	EX	
6.12	Asthma and COPD often coexist	FiMSD	2019	EX	
6.13	Moderately severe and severe COPD are often associated with comorbidities. The most important being cardiovascular diseases, metabolic syndrome, diabetes, osteoporosis, depression and numerous types of cancer.	FiMSD	2019	EX	
6.14	Anxiety and depression are major contributors to hospital bed usage and readmissions, and should be optimally managed.	COPD-X	2017	EX	
6.15	Osteoporotic fractures are a common problem in COPD due to risk factors, including smoking, physical inactivity, malnutrition, systemic inflammation, frequent use of corticosteroids, low body mass index, hypogonadism, and vitamin D deficiency. Bone mineral density testing is important for prevention and monitoring response to therapy.	COPD-X	2017	EX	

6.16	Hypoxaemia can lead to pulmonary hypertension and eventually right heart failure, particularly if there is coexisting obstructive sleep apnoea. When suspected clinically, arterial blood gases or a sleep study should be considered, with a view to oxygen therapy or continuous positive airway pressure.	COPD-X	2017	EX	
6.17	Comorbidities of COPD require identification and appropriate management	COPD-X	2017	EX	
6.18	All patients with a history of COPD should be screened for cardiovascular risk factors.	MoHS	2018	EX	
6.19	The presence of comorbidities should not alter COPD treatment and comorbidities should be treated as if the patient did not have COPD.	MoHS	2018	EX	
6.20	Anxiety and depression accompany dyspnoea and should be evaluated and treated accordingly. Benzodiazepines, tricyclic antidepressants and major tranquilisers may be useful in this context.	MoHS	2018	EX	
6.21	Assessment of comorbidities should be performed for patients diagnosed with COPD	MoHS	2018	EX	
6.22	Routinely monitor and adequately treat comorbidities in all COPD patients. Treatment of comorbidities should not change COPD management	SwRC	2018	EX	
**7**	**Referral**				
7.1	In patients under the age of 40, COPD, for example due to alpha-1 antitrypsin deficiency or abuse of (hard) drugs by inhalation, is rare. A referral to the pulmonologist is recommended for this.	NHG<br>COPD-X	2015<br>2017	IN	/<br>/
7.2	The presence of one or more alarm symptoms (exhaustion, cyanosis or a decrease in consciousness whether or not in combination with a decrease in oxygen saturation < 92%, this cut-off value does not apply to pre-existently lower saturation values) is a reason for an urgent referral.	NHG	2015	ME	/
7.3	Reasons for referral: Unusual symptoms such as haemoptysis; Frequent chest infections (ie, more than annually); SpO2 < 92% when stable	COPD-X	2017	ME	/
7.4	Refer a patient with a severe exacerbation if: no improvement occurs within half an hour; insufficient care options at home; severe interfering comorbidity; hospitalization was always necessary for previous exacerbations	NHG	2015	EX	
7.5	Referral to the pulmonologist is recommended in case of diagnostic problems or if treatment goals are not or insufficiently achieved	NHG	2015	EX	

7.6	Referral to the pulmonologist is advised in the following specific situations: in case of doubt about the diagnosis (for example if there is a discrepancy between the severity of the symptoms and objective findings), COPD at a relatively young age (arbitrary < 50 years), persistent doubt between COPD and heart failure, severe exacerbation (if there are alarm symptoms, no improvement within half an hour, insufficient care options at home, a serious interfering comorbidity or if hospitalization was always necessary in previous exacerbations), patients with a persistent moderate or severe burden of disease, a reduced nutritional status if this appears to be the result of COPD, work-related problems or obstacles	NHG	2015	EX	
7.7	Referral is desired in the following situations: two or more exacerbations per year for which the patient was treated with oral corticosteroids with or without antimicrobials, despite treatment with a long-acting bronchodilator and ICS; persistent relevant symptoms and limitations despite treatment (arbitrary MRC ≥ 3 or CCQ ≥ 2); complicating or severe comorbidity; if intensive counseling is desired due to complicating psychosocial factors that continue to make adequate treatment structural difficult; moderate to severe problems with dealing with the disease; FEV1 < 50% of predicted or < 1.5L, despite optimal treatment; fast progressive course (increase in dyspnea, decrease in exercise capacity, decrease in FEV1 over at least 3 years with on average more than 150 ml/year) despite maximum treatment, even with FEV1 > 50% of the predicted value; a possible indication for oxygen treatment such as hypoxemia	NHG	2015	EX	
7.8	The pulmonologist refers the patient to the general practitioner if the question from the general practitioner or patient has been answered, the diagnosis in the second line has been completed or the care of the pulmonologist does not further contribute to reducing the burden of disease. When referring back, the pulmonologist draws up a clear medication and non-medication policy and provides the general practitioner with relevant data with regard to the expected course of the disease, medication, degree of disease burden and applied treatment.	NHG	2015	EX	
7.9	In case of reduced nutritional status or undesirable weight loss in patients for whom a cause other than COPD is unlikely, the general practitioner will consider referral to the pulmonologist for further analysis and treatment advice	NHG	2015	EX	

7.10	Patients with a moderate or severe burden of disease who continue to experience limitations and participation problems despite optimal medical and physiotherapy care, it is recommended to discuss a rehabilitation program by referring to the pulmonologist	NHG	2015	EX	
7.11	COPD purely on the basis of other risk factors, such as chronic exposure to particulate matter or other substances in working conditions, is rare. For this purpose, referral to the pulmonologist and, if it concerns working conditions, to the medical officer is recommended.	NHG	2015	EX	
7.12	Upon referral, the general practitioner formulates an adequate question and indicates in a structured way whether it concerns a diagnostic or treatment problem and whether it involves short-term or long-term support	NHG	2015	EX	
7.13	A further analysis, usually by a pulmonologist, is advised under the following circumstances: diagnostic problems; failure or insufficient achievement of treatment goals despite adequate therapy; or patient's wish. A closer analysis should include attention to differential diagnostic problems, adaptation problems, hyperinflation, gas transport disorders and comorbidity.	LAN	2016	EX	
7.14	Re-referral according to burden of disease: Slight disease burden: re-referral to care close to home (usually after a diagnostic consultation) within 3 months; Moderate disease burden: stable and the treatment goals have since been achieved. re-referral after follow-up 3-12 months to care close to home. Unstable and the treatment goals were not achieved: second-line treatment (pulmonologist and, for example, outpatient pulmonary rehabilitation) or shared care; Severe disease burden: second-line treatment and follow-up or intensive multidisciplinary lung rehabilitation, shared care in exacerbations. If the treatment goals have been achieved after multidisciplinary lung rehabilitation, re-referral to the pulmonologist. For terminal care, a referral to care close to home can be chosen in consultation with the patient.	LAN	2016	EX	
7.15	Confusion, instable haemodynamics, oxygen saturation below 90%, pneumonia or other severe disease (e.g. diabetes, heart disease or renal failure) suggest a need for hospital treatment.	FiMSD	2019	EX	
7.16	Surgical treatment can be considered in carefully selected patients with severe COPD	FiMSD	2019	EX	

7.17	Indications for hospitalisation: Marked increase in intensity of symptoms and patient has an exacerbation characterised by increased dyspnoea, cough or sputum production, plus one or more of the following: inadequate response to appropriate community-based management; inability to walk between rooms when previously mobile; inability to eat or sleep because of dyspnoea; cannot manage at home even with homecare resources; high-risk comorbid condition (pulmonary or non-pulmonary); altered mental status suggestive of hypercapnia; worsening hypoxaemia or cor pulmonale; newly occurring arrhythmia; or newly occurring hypoxaemia (SpO2 < 92%)	COPD-X	2017	EX	
7.18	Reasons for referral: Diagnostic uncertainty and exclusion of asthma; Rapid decline in functional performance ; Persistent symptoms; Onset of ankle oedema; Assessing suitability for pulmonary rehabilitation, if uncertain; Bullous lung disease on CXR or CT; Persistent dyspnoea, marked hyperinflation, severe airflow limitation or emphysema (refer for assessment for lung transplantation, or bronchoscopic or surgical lung volume reduction procedures); Dyspnoea associated with chest tightness, anxiety or dizziness (refer for consideration of dysfunctional breathing); Daytime sleepiness, complaints by partner of heavy snoring	COPD-X	2017	EX	
**8**	**Follow-up**				
8.1	COPD patients should be followed up every 3-6 months. The CAT score should be used at each visit to track symptoms related to COPD.	MoHS	2018	IN	/
8.2	Routine yearly chest X-rays are not required.	MoHS	2018	IN	/
8.3	In the event of a serious exacerbation, a check-up is agreed the following day, which consists of: evaluation of the symptoms and experienced limitations; examination of the lungs; investigating the cause of the exacerbation (pay particular attention to therapy compliance, inhalation technique and luxating factors); possibly adjust the management	NHG	2015	EX	
8.4	Diagnosis of COPD should be accompanied by regular assessment of severity	COPD-X	2017	EX	
8.5	Check maintenance therapy and understanding	GOLD	2019	EX	
8.6	Reassess inhaler technique	GOLD	2019	EX	
8.7	Ensure understanding of withdrawal of acute medications (steroids and/or antibiotics)	GOLD	2019	EX	
8.8	Assess need for continuing any oxygen therapy	GOLD	2019	EX	
8.9	Provide management plan for comorbidities and follow-up	GOLD	2019	EX	
8.10	Ensure follow-up arrangements: early follow-up < 4 weeks, and late follow-up < 12 weeks as indicated	GOLD	2019	EX	

8.11	Follow up pharmacological management should be guided by the principles of first review and assess, then adjust if needed: review symptoms (dyspnea) and exacerbation risk, assess inhaler technique and adherence, and the role of non-pharmacological approaches and adjust pharmacological treatment, including escalation or de-escalation. Switching inhaler device or molecules within the same class may be considered as appropriate. Any change in treatment requires a subsequent review of the clinical response, including side effects.	GOLD	2019	EX	
8.12	Do not use a multidimensional index (such as BODE) to assess prognosis in people with stable COPD.	NICE	2018	EX	
8.13	At diagnosis and at each review appointment, offer people with COPD and their family members or carers (as appropriate): written information about their condition and opportunities for discussion with a healthcare professional who has experience in caring for people with COPD. At minimum, the information should cover: an explanation of COPD and its symptoms, advice on quiting smoking (if relevant) and how this will help the person's COPD, advice on avoiding passive smoke exposure, managing breathlessness, physical activity and pulmonary rehabilitation, medicines (including inhaler technique and the importance of adherence), vaccinations, identifying and managing exacerbations, details of local and national organisations and online resources that can provide more information and support and how COPD will affect other long-term conditions that are common in people with COPD (for example hypertension, heart disease, anxiety, depression and musculoskeletal problems)	NICE	2018	EX	
8.14	Advise people with COPD that the following factors increase their risk of exacerbations: continued smoking or relapse for ex-smokers, exposure to passive smoke, viral or bacterial infection, indoor and outdoor air pollution, lack of physical activity and seasonal variation (winter and spring)	NICE	2018	EX	
8.15	Develop an individualised self-management plan in collaboration with each person with COPD and their family members or carers	NICE	2018	EX	
8.16	Develop an individualised exacerbation action plan in collaboration with each person with COPD who is at risk of exacerbations	NICE	2018	EX	

8.17	Offer people a short course of oral corticosteroids and a short course of oral antibiotics to keep at home as part of their exacerbations action plan if: they have had an exacerbation within the last year and remain at risk of exacerbations, they understand and are confident about when and how to take these medicines and the associated benefits and harms, they know to tell their healthcare professional when they have used the medicines and to ask for replacements	NICE	2018	EX	
8.18	At all review appointments, discuss corticosteroid and antibiotic use with people who keep these medicines at home, to check that they still understand how to use them. For people who have used 3 or more courses of oral corticosteroids and/or oral antibiotics in the last year, investigate the possible reasons for this	NICE	2018	EX	
8.19	Encourage people with COPD to respond promptly to exacerbation symptoms by following their action plan, which may include: adjusting their short-acting bronchodilator therapy to treat their symptoms, taking a short course of oral corticosteroids if their increased breathlessness interferes with activities of daily living, adding oral antibiotics if their sputum changes colour and increases in volume or thickness beyond their normal day-to-day variation and telling their healthcare professional	NICE	2018	EX	
8.20	Ask people with COPD if they experience breathlessness they find frightening. If they do, consider including a cognitive behavioural component in their self-management plan to help them manage anxiety and cope with breathlessness	NICE	2018	EX	
8.21	For people at risk of hospitalisation, explain to them and their family members or carers (as appropriate) what to expect if this happens (including non-invasive ventilation and discussions on future treatment preferences, ceilings of care and resuscitation)	NICE	2018	EX	
8.22	Do not offer routine telehealth monitoring of physiological status as part of management for stable COPD.	NICE	2018	EX	

8.23	The frequency of monitoring after the diagnostic phase is as follows:<br>annual check without spirometry if slight disease burden (patients without symptoms and who no (longer) smoke);<br>at least an annual check and spirometry once every 3 years if slight disease burden (patients with symptoms or who smoke);<br>check at least twice a year and an annual spirometry (with adequate treatment of the condition in patients who have stopped smoking this can be done once every 3 years) if moderate burden of disease;<br>in the second line control and spirometry if serious disease burden;<br>customized care without spirometry if limited life expectancy and extra monitoring without spirometry after treatment of an exacerbation	NHG	2015	EX	
8.24	If the response to drug therapy is poor: check adherence to treatment (compliance), check that the patient has quit smoking and check correct use of the dosing device	FiMSD	2019	EX	
8.25	Patients with COPD discharged from hospital following an exacerbation should receive comprehensive follow-up led by the primary health care team	COPD-X	2017	EX	
8.26	It is recommended that elderly people diagnosed with 'light COPD' on the basis of the (canceled) criterion of an FEV1 / FVC ratio <0.7 should be reconsidered of the diagnosis of COPD by evaluating symptoms, nuisance, limitations and exacerbations and by repeating spirometry, using the GLI2012 reference values and the criterion for bronchial obstruction (FEV1 / FVC ratio <p5)	NHG	2015	EX	
8.27	Follow-up according to burden of disease: Slight disease burden: at least once a year with annual baseline assessment in primary care (case history / exacerbation frequency, MRC / CCQ / BMI / FEV1). If this assessment results in deviations, there is no longer a slight disease burden; Moderate disease burden: at least twice a year with a basic assessment at least annually (case history / exacerbation frequency, MRC / CCQ / BMI / FEV1). On indication there may be a more extensive assessment (further analysis); Severe disease burden: at least twice a year with at least annually a basic second-line assessment (case history / exacerbation frequency, MRC / CCQ / BMI / FEV1). On indication, there may be a more extensive assessment (further analysis).	LAN	2016	EX	
**9**	**Pulmonary rehabilitation**				
9.1	Pulmonary rehabilitation is indicated in all COPD patients with relevant symptoms and/or a high risk for exacerbation	GOLD	2019	ME	Evidence A
9.2	Pulmonary rehabilitation should be provided to all symptomatic COPD patients	COPD-X	2017	ME	I strong

9.3	Pulmonary rehabilitation has few adverse effects, is cost-effective and should be offered to all people with COPD who are limited by breathlessness on activity	COPD-X	2017	ME	I strong
9.4	People with stable chronic obstructive pulmonary disease (COPD) and exercise limitation due to breathlessness are referred to a pulmonary rehabilitation programme	NICE	2016	ME	/
9.5	Pulmonary rehabilitation is one of the key recommended approaches in the treatment of COPD. It should be considered for most patients with COPD, although it is especially effective in patients with moderate to severe disease. Programmes lasting 6-8 weeks are recommended for optimal benefit	SwRC	2018	ME	Evidence A
9.6	People admitted to hospital for an acute exacerbation of chronic obstructive pulmonary disease (COPD) start a pulmonary rehabilitation programme within 4 weeks of discharge.	NICE	2016	IN	/
9.7	Pulmonary rehabilitation improves symptoms, quality of life, and physical and emotional participation in everyday activities.	GOLD	2019	EX	
9.8	Programmes comprise individualised exercise programmes and education, and: are at least 6 weeks in duration and include a minimum of twice-weekly supervised sessions, include supervised, individually tailored and prescribed, progressive exercise training including both aerobic and resistance training and include a defined, structured education programme	NICE	2016	EX	
9.9	Pulmonary rehabilitation is not suitable for people with unstable cardiac disease, locomotor or neurological difficulties precluding exercise such as severe arthritis or peripheral vascular disease, and people in a terminal phase of an illness or with significant cognitive or psychiatric impairment.	NICE	2016	EX	
9.10	If a patient does not manage to move sufficiently, for example due to persistent (fear of) dyspnea, the GP will consider referring a patient with a currently moderate burden of disease to a physical therapist for exercise training	NHG	2015	EX	
9.11	Breathing exercises may be useful if shortness of breath persist, and if necessary with instruction by a physiotherapist specialized in lung reactivation	NHG	2015	EX	
9.12	If problems of mucus clearance persist, consider referring to an exercise or physical therapist	NHG	2015	EX	
9.13	Breathing exercises, pursed lip breathing and posture advice are physical therapy interventions that have a proven beneficial effect on dyspnoea and on the quality of life	LAN	2016	EX	

9.14	COPD patients, who are in a medically stable situation, are able to live independently, do not have major psychosocial and medical comorbidities and do not have complex adaptation problems, follow a basic pulmonary rehabilitation program. This is a program in which patients increase their physical condition, supplemented with a general education program, usually focused on medication use and lifestyle.	LAN	2016	EX	
9.15	COPD patients with a serious burden of disease (complex medical problems, complex adaptation problems or a combination of both) follow more intensive variants of pulmonary rehabilitation in the second or third line.	LAN	2016	EX	
9.16	Home calls made by a rehabilitation instructor are an essential part of the monitoring of patients receiving oxygen therapy at home	FiMSD	2019	EX	
9.17	As pulmonary rehabilitation reduces readmission rates and improves quality of life, the patient should be referred to pulmonary rehabilitation as soon as the acute instability has resolved.	COPD-X	2017	EX	
9.18	Consider pulmonary rehabilitation at any time, including during the recovery phase following an exacerbation	COPD-X	2017	EX	
9.19	Inpatient pulmonary rehabilitation should be started once the patient is medically stable after acute exacerbation of COPD	MoHS	2018	EX	
**10**	**Palliative and end-of-life care**				
10.1	COPD patients with FEV1 ≤ 30% and starting on long-term oxygen therapy are candidates for end-of-life discussion and Advance Care Planning	MoHS	2018	IN	Grade D, Level 4
10.2	Palliative approaches are effective in controlling symptoms in advanced COPD.	GOLD<br>COPD-X	2019<br>2017	EX	
10.3	All clinicians managing patients with COPD should be aware of the effectiveness of palliative approaches to symptom control and use these in their practice	GOLD	2019	EX	
10.4	End of life care should include discussions with patients and their families about their views on resuscitation, advance directives and place of death preferences	GOLD	2019	EX	
10.5	It is recommended that the main practitioner, in good mutual consultation and at a calm and suitable moment for the patient, discuss what is or what is not longer medically useful	NHG	2015	EX	

10.6	Whether or not a COPD patient is in the palliative phase is determined by (the deterioration in) the clinical aspect, having undergone intensive treatments without (lasting) effect and the subjective assessment and wishes of the patient and the assessment of care providers. In addition to general matters relating to the quality of life, patients are and, if possible and insofar as desired with their immediate environment, discussed and assisted with: anxiety, shame, guilt, being chronically ill, severe shortness of breath, sputum retention, questions about CPR, ventilation / treatment in IC, end-of-life care, death, oxygen therapy and comorbidity such as depression and insomnia.	LAN	2016	EX	
10.7	SSRI's can have a place in the palliative phase. Benzodiazepines can also be prescribed, especially when there is anxiety or insomnia.	LAN	2016	EX	
10.8	Opioids are effective drugs to combat shortness of breath if given orally or parenterally. Restraint when starting opioids in the palliative phase is not necessary and leads to a strong deterioration in the quality of life due to the experienced severe shortness of breath.	LAN	2016	EX	
10.9	The use of oxygen therapy usually does not lead to an improvement in dyspnoea feeling or quality of life. Nevertheless, oxygen therapy can be considered.	LAN	2016	EX	
10.10	In patients with advanced COPD, palliative care services improve symptom control and manage psychosocial and spiritual concerns. Discussion regarding advanced care directives should be undertaken as part of usual management at a suitable time in the disease course.	COPD-X	2017	EX	
10.11	Accurate assessment of approaching end of life is difficult	COPD-X	2017	EX	
10.12	Supportive, palliative and end-of-life care are beneficial for patients with advanced disease	COPD-X	2017	EX	
10.13	Clinicians who care for patients with chronic or advanced respiratory diseases should be trained in and be capable of providing basic palliative care to prevent and relieve suffering by controlling symptoms	MoHS	2018	EX	
10.14	Clinicians should consult with palliative care specialists as appropriate for managing palliative care situations beyond their level of competence	MoHS	2018	EX	
10.15	COPD patients with two or more of the following criteria are candidates for end-of-life discussion and Advance Care Planning: repeated admissions for COPD exacerbation, unintended progressive weight loss or cachexia, functional decline, development of significant comorbidities, a positive answer to the 'surprise' question "Will you be surprised if your patient dies in the next one year?" and lack of additional treatment options	MoHS	2018	EX	

Supplementary Table 3

**Definition**						
	**Recommendation**	**Source**	**Year**	**Level of evidence**	**Score**<br>(1 being the lowest score and 9 the highest score)	**Number of prioritization**
1	For all COPD patients, a classification of severity of airflow limitation is indicated as follows (in patients with $FEV_1/FVC < 0.7$):<br>GOLD 1, mild, if $FEV_1 \geq 80\%$ predicted;<br>GOLD 2, moderate, if $50\% \leq FEV_1 < 80\%$ predicted;<br>GOLD 3, severe, if $30\% \leq FEV_1 < 50\%$ predicted and<br>GOLD 4, very severe, if $FEV_1 < 30\%$ predicted	GOLD<br>SwRC	2019<br>2018	No grade<br>No grade	**1 2 3 4 5 6 7 8 9**<br>☐Not assessable<br>☐EHR extractable	
2	For all COPD patients, the assessment of symptoms and history of moderate and severe exacerbations results in a letter from A to D and should be recorded. This allows clinicians to initiate a treatment plan.	GOLD<br>MoHS	2019<br>2018	No grade<br>Grade D, Level 4	**1 2 3 4 5 6 7 8 9**<br>☐Not assessable<br>☐EHR extractable	
**Remarks or suggestions for new recommendations:**						
**Screening**						
	**Recommendation**	**Source**	**Year**	**Level of evidence**	**Score**<br>(1 being the lowest score and 9 the highest score)	**Number of prioritization**
3	COPD should be considered in any patient who has dyspnoea (progressive, on exertion or persistent), chronic cough or sputum production, a history of recurrent lower respiratory tract infections and/or a history of exposure to risk factors (e.g., tobacco smoking).	GOLD<br>NHG<br>LAN<br>COPD-X<br>MoHS<br>SwRC<br>NICE	2019<br>2015<br>2016<br>2017<br>2018<br>2018<br>2016	No grade<br>No grade<br>No grade<br>No grade<br>Grade D, Level 4<br>No grade<br>No grade	**1 2 3 4 5 6 7 8 9**<br>☐Not assessable<br>☐EHR extractable	

4	Screening spirometry in the general asymptomatic population is not recommended.	MoHS<br>USPSTF	2018<br>2016	Grade D, Level 4<br>Grade D	**1 2 3 4 5 6 7 8 9**<br>☐Not assessable<br>☐EHR extractable	
5	Clinicians should ask all adults, including pregnant women, about tobacco use.	USPSTF	2016	Grade D	**1 2 3 4 5 6 7 8 9**<br>☐Not assessable<br>☐EHR extractable	
**Remarks or suggestions for new recommendations:**						

Diagnosis

Physical examination and technical investigations

	**Recommendation or QI**	**Source**	**Year**	**Level of evidence**	**Score** (1 being the lowest score and 9 the highest score)	**Number of prioritization**
6	% COPD patients with registration BMI and 'unwanted weight loss' in the last twelve months	Cebam	2018	No grade	**1 2 3 4 5 6 7 8 9**<br>☐Not assessable<br>☐EHR extractable	
7	% COPD patients GOLD 4 and a measurement of the oxygen saturation in the last twelve months	Cebam	2019	No grade	**1 2 3 4 5 6 7 8 9**<br>☐Not assessable<br>☐EHR extractable	
8	Spirometry is required to make the diagnosis. The presence of a post-bronchodilator $FEV_1/FVC < 0.70$ confirms persistent airflow limitation.	GOLD<br>COPD-X<br>FiMSD<br>SwRC<br>MoHS	2019<br>2017<br>2019<br>2018<br>2018	No grade<br>III-2 strong<br>No grade<br>No grade<br>Grade D, Level 4	**1 2 3 4 5 6 7 8 9**<br>☐Not assessable<br>☐EHR extractable	
9	Spirometry is required to make the diagnosis. A FEV_1/FVC < 5th percentile of the reference population (or $z < -1.65$) after bronchodilation is consistent with COPD.	NHG<br>FiMSD	2015<br>2019	No grade<br>No grade	**1 2 3 4 5 6 7 8 9**<br>☐Not assessable<br>☐EHR extractable	

10	In case of abnormal spirometry, repeat the test after 6 weeks, so that the patient is recovered from a possibly first presented exacerbation of COPD.	NHG	2015	No grade	**1 2 3 4 5 6 7 8 9**<br>☐Not assessable<br>☐EHR extractable	
**Remarks or suggestions for new recommendations:**						
**Treatment**						
Prevention and nonpharmacological						
	**Recommendation or QI**	**Source**	**Year**	**Level of evidence**	**Score**<br>(1 being the lowest score and 9 the highest score)	**Number of prioritization**
11	Smoking cessation is recommended for all COPD patients.	GOLD<br>COPD-X<br>NHG<br>FiMSD<br>MoHS<br>SwRC<br>USPSTF	2019<br>2017<br>2015<br>2019<br>2018<br>2018<br>2016	Evidence A<br>II strong<br>No grade<br>No grade<br>Grade A, Level 1<br>Evidence A<br>Grade D	**1 2 3 4 5 6 7 8 9**<br>☐Not assessable<br>☐EHR extractable	
12	All patients with COPD should have an annual influenza vaccination.	GOLD<br>COPD-X<br>NHG<br>LAN<br>FiMSD<br>MoHS<br>SwRC	2019<br>2017<br>2015<br>2016<br>2019<br>2018<br>2018	Evidence B<br>I strong<br>No grade<br>No grade<br>No grade<br>Grade A, Level 1<br>Evidence B	**1 2 3 4 5 6 7 8 9**<br>☐Not assessable<br>☐EHR extractable	
13	All patients ≥ 65 years with COPD and all younger patients with significant comorbid conditions (including chronic heart or lung disease) should receive the pneumococcal vaccination: the PCV13 and PPSV23.	GOLD<br>COPD-X<br>FiMSD<br>MoHS<br>SwRC	2019<br>2017<br>2019<br>2018<br>2018	Evidence B<br>I strong<br>No grade<br>Grade C, Level 2<br>No grade	**1 2 3 4 5 6 7 8 9**<br>☐Not assessable<br>☐EHR extractable	

14	Supplemental oxygen therapy should be prescribed to stable COPD patients if Pa0$_2$ ≤ 55 mmHg or SaO_2 < 88%.	GOLD<br>MoHS<br>SwRC	2019<br>2018<br>2018	Evidence A<br>Grade A, Level 1<br>Evidence A	**1 2 3 4 5 6 7 8 9**<br>☐Not assessable<br>☐EHR extractable	
15	% COPD patients in whom degree of physical activity is determined	Cebam	2018	No grade	**1 2 3 4 5 6 7 8 9**<br>☐Not assessable<br>☐EHR extractable	

Remarks or suggestions for new recommendations:

Pharmacological

<u>Maintenance therapy</u>

Inhaled therapy: bronchodilators + ICS

	**<u>Recommendation</u>**	**<u>Source</u>**	**<u>Year</u>**	**<u>Level of evidence</u>**	**<u>Score</u>**<br>(1 being the lowest score and 9 the highest score)	**<u>Number of prioritization</u>**
16	For patients with COPD, inhaled bronchodilators are preferred over oral bronchodilators. Bronchodilators are recommended as the initial treatment for all COPD groups.	GOLD<br>SwRC<br>LAN	2019<br>2018<br>2016	Evidence A<br>Evidence A<br>No grade	**1 2 3 4 5 6 7 8 9**<br>☐Not assessable<br>☐EHR extractable	
17	For patients with COPD, LABAs and LAMAs are preferred over short-acting agents except for patients with only occasional dyspnoea, and for immediate relief of symptoms in patients already on long-acting bronchodilators for maintenance therapy.	GOLD<br>COPD-X<br>MoHS<br>SwRC	2019<br>2017<br>2018<br>2018	Evidence A<br>No grade<br>Grade A, Level 1<br>Evidence A	**1 2 3 4 5 6 7 8 9**<br>☐Not assessable<br>☐EHR extractable	

18	Rescue short-acting bronchodilators should be prescribed to all patients for immediate symptom relief.	GOLD	2019	No grade	**1 2 3 4 5 6 7 8 9**<br>☐Not assessable<br>☐EHR extractable	
19	All group A patients should be offered a short- or a long-acting bronchodilator.	GOLD	2019	No grade	**1 2 3 4 5 6 7 8 9**<br>☐Not assessable<br>☐EHR extractable	
20	For group B, initial therapy should consist of a LABA or a LAMA. Patients with persistent breathlessness should be escalated to a LABA/LAMA combination.	GOLD<br>MoHS<br>SwRC<br>COPD-X	2019<br>2018<br>2018<br>2017	Evidence A<br>Grade A, Level 1<br>Evidence A<br>No grade	**1 2 3 4 5 6 7 8 9**<br>☐Not assessable<br>☐EHR extractable	
21	For group C patients, starting therapy with a LAMA is recommended.	GOLD<br>MoHS	2019<br>2018	No grade<br>Grade A, Level 1	**1 2 3 4 5 6 7 8 9**<br>☐Not assessable<br>☐EHR extractable	
22	For patients in group C, a second long-acting bronchodilator is the preferred treatment option if exacerbations persist.	SwRC<br>MoHS	2018<br>2018	Evidence A<br>Grade A, Level 1	**1 2 3 4 5 6 7 8 9**<br>☐Not assessable<br>☐EHR extractable	
23	Patients in group D should be started on a LABA/LAMA combination, guided by the level of symptoms (e.g. CAT > 20).	GOLD<br>MoHS	2019<br>2018	No grade<br>Grade A, Level 1	**1 2 3 4 5 6 7 8 9**<br>☐Not assessable<br>☐EHR extractable	

24	For patients with persistent exacerbations on long-acting bronchodilator monotherapy, escalation to either LABA/LAMA or LABA/ICS is recommended.	GOLD<br>MoHS	2019<br>2018	No grade<br>Grade A, Level 1	**1 2 3 4 5 6 7 8 9**<br>☐Not assessable<br>☐EHR extractable	
25	In patients who develop further exacerbations on LABA/LAMA therapy two alternative pathways are suggested: escalation to LABA/LAMA/ICS if blood eosinophil counts ≥ 100 cells/µL or add roflumilast or azithromycin if blood eosinophil < 100 cells/µL	GOLD	2019	No grade	**1 2 3 4 5 6 7 8 9**<br>☐Not assessable<br>☐EHR extractable	
26	In patients who develop further exacerbations on LABA/ICS therapy, escalation to triple therapy by adding a LAMA is recommended.	GOLD	2019	No grade	**1 2 3 4 5 6 7 8 9**<br>☐Not assessable<br>☐EHR extractable	
**Remarks or suggestions for new recommendations:**						

Oral therapy: antibiotics, methylxanthines, and mucolytic agents

	**Recommendation**	**Source**	**Year**	**Level of evidence**	**Score**<br>(1 being the lowest score and 9 the highest score)	**Number of prioritization**
27	Before starting azithromycin, ensure the patient has had: an electrocardiogram (ECG) to rule out prolonged QT interval and baseline liver function tests. Review prophylactic azithromycin after the first 3 months, and then at least every 6 months.	NICE	2018	No grade	**1 2 3 4 5 6 7 8 9**<br>☐Not assessable<br>☐EHR extractable	

28	Theophylline is not recommended unless other long-term treatment bronchodilators are unavailable or unaffordable.	GOLD<br>SwRC	2019<br>2018	Evidence B<br>Evidence B	**1 2 3 4 5 6 7 8 9**<br>☐Not assessable<br>☐EHR extractable	
29	Mucolytic drug therapy should be considered in patients with a chronic productive cough and should be continued if there is symptomatic improvement.	MoHS	2018	Grade B, Level 1	**1 2 3 4 5 6 7 8 9**<br>☐Not assessable<br>☐EHR extractable	
**Remarks or suggestions for new recommendations:**						

Therapy for an acute exacerbation

	Recommendation	Source	Year	Level of evidence	Score (1 being the lowest score and 9 the highest score)	Number of prioritization
30	To treat an acute exacerbation, a SABA with or without a SAMA should be used as initial bronchodilator. For example, increased doses of salbutamol 4-8 puffs via a metered dose inhaler and spacer every 3-4 hours should be used.	GOLD<br>SwRC<br>MoHS<br>COPD-X	2019<br>2018<br>2018<br>2017	Evidence C<br>Evidence C<br>Grade C, Level 2<br>I strong	**1 2 3 4 5 6 7 8 9**<br>☐Not assessable<br>☐EHR extractable	
31	To treat an acute exacerbation of COPD, methylxanthines should not be used.	GOLD<br>SwRC	2019<br>2018	Evidence B<br>Evidence B	**1 2 3 4 5 6 7 8 9**<br>☐Not assessable<br>☐EHR extractable	
32	To manage an exacerbation of COPD with a significant increase in breathlessness which interferes with daily activities, offer 30 mg oral prednisone daily. Glucocorticosteroid treatment should not be given for more than 5-7 days.	NICE<br>MoHS<br>SwRC<br>COPD-X	2018<br>2018<br>2018<br>2017	No grade<br>Grade A, Level 1<br>Evidence A<br>I strong	**1 2 3 4 5 6 7 8 9**<br>☐Not assessable<br>☐EHR extractable	

33	For exacerbations with signs and symptoms of infection (increased volume and change in colour of sputum or fever), antibiotics should be offered. First-line agents include oral amoxicillin or doxycycline for 5-7 days.	COPD-X<br>MoHS<br>SwRC	2017<br>2018<br>2018	II strong<br>Grade A, Level 1<br>Evidence B	**1 2 3 4 5 6 7 8 9**<br>☐Not assessable<br>☐EHR extractable	
**Remarks or suggestions for new recommendations:**						

Referral

	Recommendation	Source	Year	Level of evidence	Score (1 being the lowest score and 9 the highest score)	Number of prioritization
34	Patients under the age of 40 with COPD should be referred to a pulmonologist.	NHG<br>COPD-X	2015<br>2017	No grade<br>No grade	**1 2 3 4 5 6 7 8 9**<br>☐Not assessable<br>☐EHR extractable	
35	Referral is indicated in the following circumstances:<br>- SpO_2 < 92% when stable<br>- Haemoptysis<br>- Frequent chest infections (i.e., more than [illegible]	NHG<br>COPD-X	2015<br>2017	No grade<br>No grade	**1 2 3 4 5 6 7 8 9**<br>☐Not assessable<br>☐EHR extractable	
**Remarks or suggestions for new recommendations:**						

Follow-up

	Recommendation or QI	Source	Year	Level of evidence	Score (1 being the lowest score and 9 the highest score)	Number of prioritization

36	COPD patients should be followed up every 3-6 months. The CAT score should be used at each visit to track symptoms related to COPD.	MoHS	2018	No grade	**1 2 3 4 5 6 7 8 9**<br>☐Not assessable<br>☐EHR extractable	
37	Routine yearly chest X-rays are not required.	MoHS	2018	No grade	**1 2 3 4 5 6 7 8 9**<br>☐Not assessable<br>☐EHR extractable	
38	% COPD patients with an indication of the number of exacerbations in the last twelve months and an assessment of the dyspnoea using the mMRC score	Cebam	2019	No grade	**1 2 3 4 5 6 7 8 9**<br>☐Not assessable<br>☐EHR extractable	
39	% COPD patients in whom the degree of functioning using the mMRC score is determined	Cebam	2018	No grade	**1 2 3 4 5 6 7 8 9**<br>☐Not assessable<br>☐EHR extractable	
**Remarks or suggestions for new recommendations:**						

Pulmonary rehabilitation

	Recommendation	Source	Year	Level of evidence	Score<br>(1 being the lowest score and 9 the highest score)	Number of prioritization

40	Pulmonary rehabilitation is indicated in all COPD patients with relevant symptoms and/or a high risk for exacerbations.	GOLD<br>COPD-X<br>NICE<br>SwRC	2019<br>2017<br>2016<br>2018	Evidence A<br>I strong<br>No grade<br>Evidence A	**1 2 3 4 5 6 7 8 9**<br>☐Not assessable<br>☐EHR extractable	
41	Patients hospitalized for a COPD exacerbation, initiate a pulmonary rehabilitation program within 4 weeks of discharge.	NICE	2016	No grade	**1 2 3 4 5 6 7 8 9**<br>☐Not assessable<br>☐EHR extractable	

Remarks or suggestions for new recommendations:

Palliative and end-of-life care

	Recommendation	Source	Year	Level of evidence	Score (1 being the lowest score and 9 the highest score)	Number of prioritization
42	COPD patients with FEV_1 ≤ 30% and starting on long-term oxygen therapy are candidates for end-of-life discussion and Advance Care Planning.	MoHS	2018	Grade D, Level 4	**1 2 3 4 5 6 7 8 9**<br>☐Not assessable<br>☐EHR extractable	

Remarks or suggestions for new recommendations:
