# Supplementary material for: Development of primary care quality indicators for chronic obstructive pulmonary disease using a Delphi-derived method
Source: NPJ Prim Care Respir Med. 2022 Mar 18;32:12. doi: 10.1038/s41533-022-00276-w (PMC8933430; doi:10.1038/s41533-022-00276-w)
Supplement: Supplementary file 2 — Reporting Summary [file 41533_2022_276_MOESM2_ESM.pdf]

nature portfolio

Corresponding author(s): Steve Van den Bulck
steve.vandenbulck@kuleuven.be

Last updated by author(s): Dec 8, 2021

Reporting Summary

Nature Portfolio wishes to improve the reproducibility of the work that we publish. This form provides structure for consistency and transparency in reporting. For further information on Nature Portfolio policies, see our Editorial Policies and the Editorial Policy Checklist.

Statistics

For all statistical analyses, confirm that the following items are present in the figure legend, table legend, main text, or Methods section.

n/a	Confirmed	
☐	☒	The exact sample size (n) for each experimental group/condition, given as a discrete number and unit of measurement
☐	☒	A statement on whether measurements were taken from distinct samples or whether the same sample was measured repeatedly
☐	☒	The statistical test(s) used AND whether they are one- or two-sided<br>*Only common tests should be described solely by name; describe more complex techniques in the Methods section.*
☒	☐	A description of all covariates tested
☒	☐	A description of any assumptions or corrections, such as tests of normality and adjustment for multiple comparisons
☒	☐	A full description of the statistical parameters including central tendency (e.g. means) or other basic estimates (e.g. regression coefficient) AND variation (e.g. standard deviation) or associated estimates of uncertainty (e.g. confidence intervals)
☒	☐	For null hypothesis testing, the test statistic (e.g. F, t, r) with confidence intervals, effect sizes, degrees of freedom and P value noted<br>*Give P values as exact values whenever suitable.*
☒	☐	For Bayesian analysis, information on the choice of priors and Markov chain Monte Carlo settings
☒	☐	For hierarchical and complex designs, identification of the appropriate level for tests and full reporting of outcomes
☒	☐	Estimates of effect sizes (e.g. Cohen's d, Pearson's r), indicating how they were calculated

Our web collection on statistics for biologists contains articles on many of the points above.

Software and code

Policy information about availability of computer code

Data collection	We searched for national and international guidelines in the World Wide Web and in the literature database MEDLINE. These guidelines had to be evidence-based and developed by internationally renowned institutions.
Data analysis	We used Excel to do the data analysis (e.g., calculation of the median Likert scale score).

For manuscripts utilizing custom algorithms or software that are central to the research but not yet described in published literature, software must be made available to editors and reviewers. We strongly encourage code deposition in a community repository (e.g. GitHub). See the Nature Portfolio guidelines for submitting code & software for further information.

Data

Policy information about availability of data

All manuscripts must include a data availability statement. This statement should provide the following information, where applicable:

- Accession codes, unique identifiers, or web links for publicly available datasets
- A description of any restrictions on data availability
- For clinical datasets or third party data, please ensure that the statement adheres to our policy

The data that support the results of this study are available from the corresponding author upon reasonable request.

Field-specific reporting

Please select the one below that is the best fit for your research. If you are not sure, read the appropriate sections before making your selection.

☒ Life sciences ☐ Behavioural & social sciences ☐ Ecological, evolutionary & environmental sciences

For a reference copy of the document with all sections, see nature.com/documents/nr-reporting-summary-flat.pdf

Life sciences study design

All studies must disclose on these points even when the disclosure is negative.

Sample size	Twelve participants were included in the panel. In a RAND-modified Delphi method, panels were preferable composed of 10-15 members.
Data exclusions	The entire selection process was based on language (English and Dutch) and year of publication (after 2012). References were retained if they were clinical guidelines or recommendation statements aimed at general practitioners in which all aspects of the care for COPD, including definition, screening, diagnosis, treatment, management, comorbidities, referral, follow-up, pulmonary rehabilitation or palliative and end-of-life care, was recommended. Also, the AGREE II instrument was used to exclude guidelines of low quality.
Replication	We described the concrete search terms for improvement of the reproducibility. We added all recommendations extracted from the included guidelines on which we built up the questionnaire in the Supplementary Data.
Randomization	Randomization is not relevant to the design of our study (a RAND-modified Delphi method with one panel).
Blinding	Blinding is not relevant to the design of our study (a RAND-modified Delphi method).

Reporting for specific materials, systems and methods

We require information from authors about some types of materials, experimental systems and methods used in many studies. Here, indicate whether each material, system or method listed is relevant to your study. If you are not sure if a list item applies to your research, read the appropriate section before selecting a response.

Materials & experimental systems

n/a	Involved in the study	
☒	☐	Antibodies
☒	☐	Eukaryotic cell lines
☒	☐	Palaeontology and archaeology
☒	☐	Animals and other organisms
☐	☒	Human research participants
☒	☐	Clinical data
☒	☐	Dual use research of concern

Methods

n/a	Involved in the study	
☒	☐	ChIP-seq
☒	☐	Flow cytometry
☒	☐	MRI-based neuroimaging

Human research participants

Policy information about studies involving human research participants

Population characteristics	We aimed to compose a multidisciplinary panel based on profession, patient involvement and expertise. All patients needed to have the diagnosis of COPD for more than 10 years and all professionals needed to treat patients with COPD on a daily basis.
Recruitment	Recruitment strategies included using our own network as general practitioners and as academic staff, sending e-mail invitations through hospitals (regional and university hospitals in two different provinces), and contacting members of a non-profit patient association. We contacted potential participants via e-mail or phone call.
Ethics oversight	The study was approved on December 9, 2019, by the Research Ethics Committee UZ/KU Leuven with reference MP012007.

Note that full information on the approval of the study protocol must also be provided in the manuscript.
